# Nonreciprocal feedback induces migrating oblique and horizontal banded vegetation patterns in hyperarid landscapes

**DOI:** 10.1038/s41598-024-63820-3

**Published:** 2024-06-25

**Authors:** Belén Hidalgo-Ogalde, David Pinto-Ramos, Marcel G. Clerc, Mustapha Tlidi

**Affiliations:** 1 Departamento de Física and Millennium Institute for Research in Optics, FCFM, Universidad de Chile, Casilla 487-3, Santiago, Chile; 2 Département de Physique, Faculté des Sciences, Université Libre de Bruxelles (U.L.B), CP 231, Campus Plaine, 1050 Brussels, Belgium; 3 Present Address: Center for Advanced Systems Understanding (CASUS), Helmholtz-Zentrum Dresden-Rossendorf (HZDR), Görlitz, Germany

**Keywords:** Applied physics, Ecology

## Abstract

In hyperarid environments, vegetation is highly fragmented, with plant populations exhibiting non-random biphasic structures where regions of high biomass density are separated by bare soil. In the Atacama Desert of northern Chile, rainfall is virtually nonexistent, but fog pushed in from the interior sustains patches of vegetation in a barren environment. Tillandsia landbeckii, a plant with no functional roots, survives entirely on fog corridors as a water source. Their origin is attributed to interaction feedback among the ecosystem agents, which have different spatial scales, ultimately generating banded patterns as a self-organising response to resource scarcity. The interaction feedback between the plants can be nonreciprocal due to the fact that the fog flows in a well-defined direction. Using remote sensing analysis and mathematical modelling, we characterise the orientation angle of banded vegetation patterns with respect to fog direction and topographic slope gradient. We show that banded vegetation patterns can be either oblique or horizontal to the fog flow rather than topography. The initial and boundary conditions determine the type of the pattern. The bifurcation diagram for both patterns is established. The theoretical predictions are in agreement with observations from remote sensing image analysis.

## Introduction

**Figure 1:**
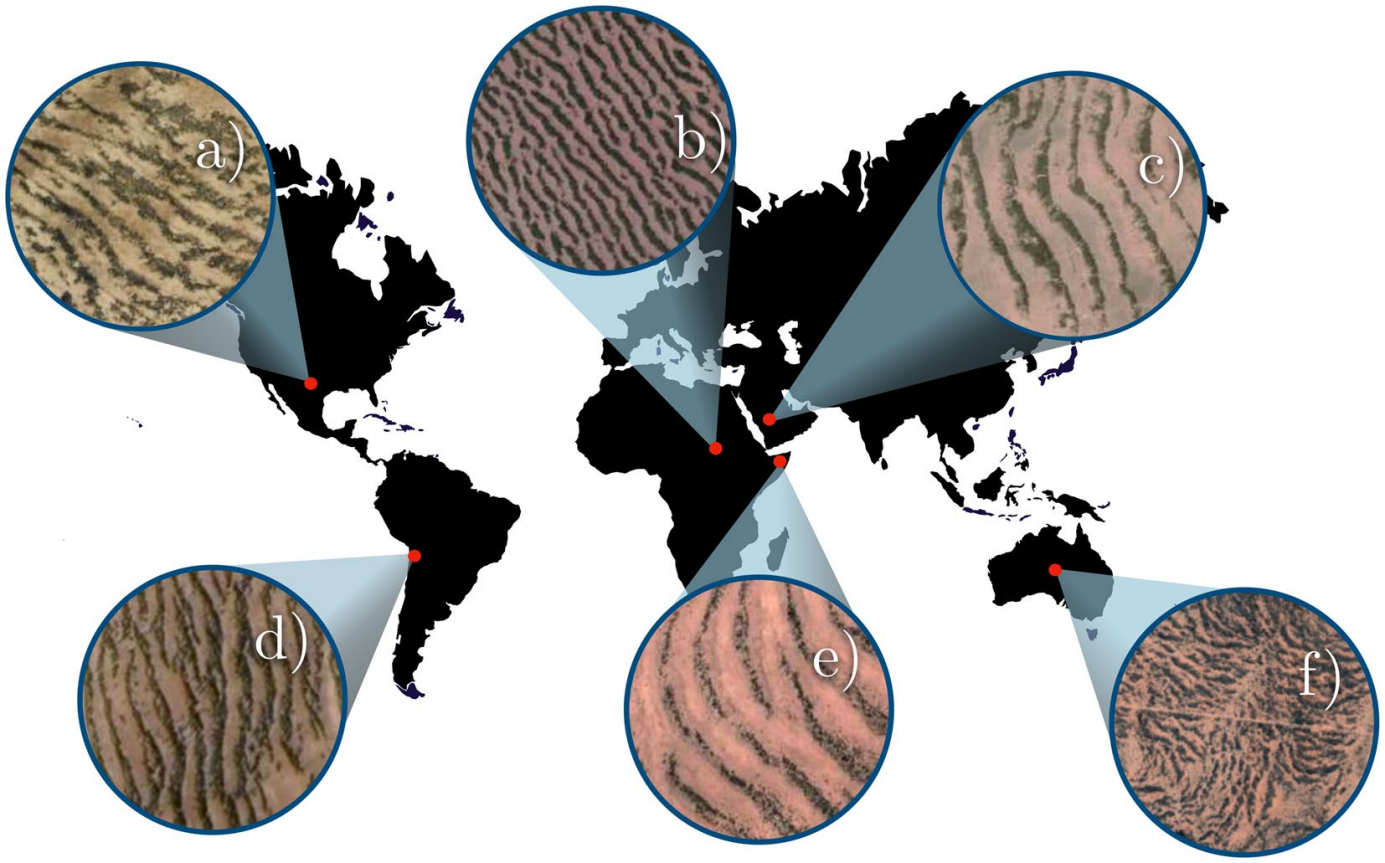
Tiger Bush patterns around the world. Vegetation banded patterns living in the coordinates: (**a**) Texas, USA, 31^∘^ 02^′^ N, 103^∘^, 06^′^ W. (**b**) West Kordofan, Sudan, 11^∘^ 20^′^ N, 28^∘^ 19^′^ E. (**c**) Saudi Arabia, 24^∘^ 19^′^ N, 42^∘^ 56^′^ E. (**d**) Atacama desert, Chile, 20^∘^ 24^′^ S, 70^∘^ 05^′^ W. (**e**) Sanaag, Somalia, 9^∘^ 48^′^ N, 48^∘^ 55^′^ E. (**f**) Northern Territory, Australia, 22^∘^ 40^′^ S, 134^∘^ 05^′^ E.

Macfayden is credited with being the first to document vegetation patterns such as bands and/or labyrinths in the early 1950s^1^. Advances in aerial photography have made it possible to make these spatial large-scale observations, often invisible from the ground. Vegetation patterns are sparsely populated or bare areas alternating with dense vegetation patches. It is often referred to them as tiger bush^2^. They have been seen throughout large areas in numerous landscape locations in Africa, America, Australia, and the Middle East^2–4^. Banded vegetation pattern includes shrubs, trees and grasses. They can grow on clay, loam, and sandy soils and are not restricted to a single soil type. They are specific to arid and semi-arid landscapes where the annual rainfall (50-750 mm) is low with regard to potential evapotranspiration (larger than $1.5 \times 10^3$ mm). As annual rainfall decreases, the average vegetation density decreases while the pattern wavelength increases^5^. Figure 1 shows examples of tiger bush. Permanent, non-transient topological defects characterize these vegetation patterns^6^. In addition to tiger bush, other types of vegetation consisting of a regular distribution of arcs^7^, patches or gaps have been reported^8^. More recently, other vegetation patterns in the form of spirals have been documented^9,10^.

Mathematical modelling of biological and ecological systems is challenging because these systems lack basic physical principles. An early discrete modelling approach based on cellular automata has been proposed^11–13^. Soon after, modelling approaches that are continuous in time and space, explaining vegetation patterns and self-organisation, proliferated. They fall into three categories. The first approach, commonly known as the interaction-redistribution model, is based on the relationship between the structure of individual plants and the facilitation-competition interactions existing within plant communities^14–18^. The second approach incorporates explicitly water transport^19^. Other reaction-diffusion models that are mainly the extensions of Klausmeier model^19^ has been proposed in the literature^20–26^. The third approach focuses on the role of environmental inhomogeneities, either in space or time, as a source of symmetry-breaking transitions induced by noise^27–31^. Despite the diversity of modelling approaches, the symmetry-breaking at the origin of the formation of periodic structures follows the generic sequence: a homogeneous cover becomes spontaneously unstable and gives rise to gaped structures, then banded pattern or labyrinthine structures, and finally spot structures before collapsing onto the bare ground as the level of aridity increases^32,33^. This sequence has been found in other reaction-diffusion type of models^20,34,35^. Analysis of remote sensing images confirms this theoretical prediction^36,37^. Vegetation patterns are not always periodic; they can be aperiodic and localised in space^38–42^. The interaction between localized gaps and patches has been documented^15,43^. However, the circular shapes of localised spots can become unstable due to the curvature instability, leading to a phenomenon of self-replication and allowing arid ecosystems to repopulate^44,45^.

It is now widely accepted that symmetry-breaking is responsible for the spontaneous formation of vegetation patterns, even under homogeneous and isotropic environmental conditions^14^. However, spatial anisotropies, such as vegetation growing on a slope, are inherent to most landscapes^46,47^. The slope of the ground alters the pattern selection process because it generates nonreciprocal feedback between plants, i.e., biomass privileges its development in certain spatial symmetry and/or orientation^14^. Patches or gaps are replaced by migrating banded patterns or arcs. More recently, remote sensing image analysis has shown that vegetation stripes form a downward convex arc when growing on the top of a ridge and an upward convex arc when growing in a valley^48^. This scenario is supported by numerical simulations of the Klausmeier model modified to include the influence of terrain curvature^48^. An upslope moving vegetation pattern was observed using a reaction-diffusion model with advection describing the coupling between biomass and toxicity^49^. The travelling vegetation patterns presented in this work are not specific to arid ecosystems. A further generalization of Klausmeier’s model with inertia and secondary seed dispersal effects has been shown to support travelling banded solutions^50^. An early explanation of an upward migration of bands has been attributed to the redistribution of water from scattered patches of vegetation to dense patches via runoff^51,52^, or dominant wind^53,54^. Models that take into account the slope of the ground as a precondition for the formation of vegetation are unable to explain the formation of regular patches and/or gaps^19^. The wavelength and the speed of horizontal uphill banded vegetation have been evaluated^55^.

The orientation of the banded vegetation, orthogonal to vegetation lines, is not always parallel to the slope. Their orientation can be orthogonal^14,56^ or even oblique, as shown by Dunkerely and Brown^57^ in arid landscapes of Australia. The proposed mechanism is mainly attributed to precipitation in sloping landscapes. Other hyperarid ecosystems such as the Atacama desert, where vegetation is constituted by *Tillandsia landbeckii* develops banded vegetation in fog-dependent environments^58–60^. These vegetation populations are devoid of root systems and survive in a hyperarid environment. The coastal region of northern Chile and southern Peru is characterised by a hyperarid climate defined by annual precipitation of less than 25 mm^61^. *Tillandsia landbeckii* catches water droplets and nutrients from the advected fog in the direction west-to-east from the Pacific Ocean^62^. The vegetation covers mainly the west side of hills, suggesting that topography plays a crucial role in spatial vegetation confinement (see Fig 7 in “Materials and methods” section).

The aim of this work is to investigate the spatiotemporal dynamics of the bands of *Tillandsia landbeckii* as a function of their orientation in relation to the topographical gradient and the dominant wind direction. For this purpose, we consider a generic interaction redistribution model with nonreciprocal feedback from neighbouring plants. Nonreciprocity means that the facilitation and competitive interactions are asymmetrical. This is because plants absorb water droplets and nutrients from moving fog. For each plant, the interaction with its front neighbour with respect to incoming fog is different from that of its rear neighbour. We show that as the aridity parameter increases, horizontal bands form, followed by the stabilisations of two oblique bands. Both horizontal and oblique bands have an overlapping domain of stability. Indeed, depending on the initial and boundary conditions, both bands can be observed. We include appropriate boundary conditions to model the behaviour of *tillandsia*, clarifying the observed patterns. Oblique vegetation bands have never been reported in any of the above-mentioned modelling approaches.

## Results

### *Tillandsia landbeckii* banded patterns: remote sensing image analysis

**Figure 2:**
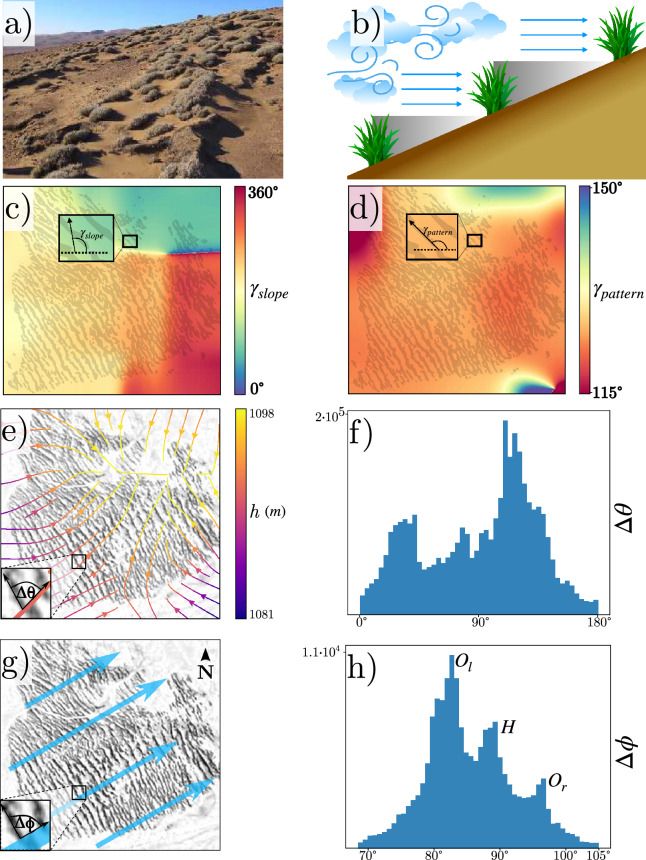
Statistical analysis of *Tillandsia landbeckii* patterns alignment. (**a**) Photograph of *Tillandsiales* in the north of Chile (courtesy of Nicolás Lavandero, some rights reserved (CC BY https://creativecommons.org/licenses/by/4.0/)). (**b**) Schematic representation of nonreciprocal feedback for fog-dependent plants. (**c**) Slope orientation map of a vegetation pattern patch, where the colours show different orientations. The inset shows the angle $\gamma _{\textit{slope}}$ generated by the slope gradient. (**d**) Angle of the banded pattern with respect to the east axis. The inset illustrates the angle, $\gamma _{\textit{pattern}}$, which the banded pattern forms with respect to the horizontal axis. (**e**) Slope gradient streamlines for a patch of *Tillandsia landbeckii*. The colours in the curves account for the height *h*. $\Delta \theta$ stands for the angle between the vegetation band and the topography gradient. (**f**) $\Delta \theta$ distribution of the banded pattern and the direction of the slope. (**g**) Representation of the direction of fog propagation (wind) for a vegetation pattern patch. $\Delta \phi$ accounts for the orientation angle between the vegetation band and wind. (**h**) $\Delta \phi$ distribution histogram between wind direction and the vegetation banded pattern. $O_l$, *H*, and $O_r$ account for the oblique left, horizontal, and oblique right pattern.

*Tillandsia* species are plants original to the coastal Peru and northern Chile areas, characterised by being epiphytic and unrooted^63^. *Tillandsia landbeckii* corresponds to a specialised epiarenic specie dominating the population of fog-dependent ecosystems in northern Chile^58,63^; their vast community formations are often called *tillandsiales*^58^. An example of such communities is shown in Fig. 2a. They are characterised by capturing fog water and having exceptional capabilities for its retention against the hyper-arid conditions of the Atacama desert^64,65^. The fog moves inland from the Pacific Ocean in a preferred direction from west to east ^59^, reaching the banded vegetation patterns living on sloped terrain as schematised in Fig. 2b.

We compute first the orientation field of the pattern $\gamma _{\textit{pattern}}$ which measures the angle with respect to a fixed horizontal direction of the banded vegetation pattern (see Fig. 2c). Second, we compute the angle with respect to the horizontal direction $\gamma _{\textit{slope}}$ which measures the angle of topographic gradient as shown in Fig. 2d (see “Materials and methods” section for details). Figure 2c illustrates how the slope direction angle $\gamma _{\textit{slope}}$ changes across the pattern with respect to the horizontal direction. Figure 2d shows a banded pattern with an approximate $120^{\circ }$, counterclockwise with respect to the east, which seems to not vary with changes in slope direction. To see the relation between these two orientations, we measure the difference angles $\Delta \theta (\textbf{r})$ between pattern orientation $\gamma _{\textit{pattern}}$ and slope $\gamma _{\textit{slope}}$ at the spatial position $\textbf{r}$, as shown in Fig. 2e. Based on typical reaction-diffusion theoretical models, one would expect a $90^\circ$ difference throughout space, that is, bands orient perpendicular to the slope. Figure 2f summarises these measurements. Unexpectedly, we obtain a distribution $\Delta \theta$ that does not exhibit a $90^\circ$ dominance. From these charts, we can infer that there is no clear correlation between the slope angle and the orientation of the banded pattern.

On the other hand, the wind’s mean direction is west to east, with a slight $30^{\circ }$ tilt counterclockwise with respect to east ^66^, as depicted in Fig. 2g, blue arrows account for the average wind propagation. Additionally, via satellite images  ^67^, we observe that, on average, bands align perpendicularly to the wind direction. To shed light on the wind effect on patterns, we locally measure the orientation angle $\Delta \phi (\textbf{r})$ between the pattern angle $\gamma _{\textit{pattern}}$ and the wind direction $\Gamma$ (see Fig. 2g), determining its angular distribution histogram (cf. Fig. 2h). By analysing several vegetation bands within vegetation pattern patches, we observe that there are deviations from the perpendicular direction, thus observing the phenomenon of *oblique bands*, as illustrated in Fig. 2h. From this chart, we infer that the distribution is trimodal, characterised by the horizontal (perpendicular to wind direction) and two oblique patterns with $\pm 7^{\circ }$ of difference from the horizontal one. Indeed, this histogram shows the coexistence of vegetation-banded patterns with different orientations. From these charts, we can infer that there is a relationship between wind propagation and the orientation of the banded pattern. Going further and calculating the correlation $\langle \cos (\gamma _{\textit{pattern}}-\Gamma )\rangle \approx 0.98$ and $\langle \cos (\gamma _{\textit{pattern}}-\gamma _{\textit{slope}})\rangle \approx 0.1$, where the symbol $\langle \rangle$ accounts for the spatial average. Noting that, the angle used to compute the correlation is perpendicular to $\gamma _{\textit{pattern}}$. For more details, check the “Material and methods” section. The results indicate that the pattern propagation direction is more aligned with the wind direction than with the slope gradient direction. Hence, this is analogous to the phenomenon of oblique vegetation patterns involving vegetation with functional roots, which could be oblique or not to the slope direction in which water flows^57^. Note that there is no histogram with perfectly symmetrical angular deviations between wind direction and band pattern due to variations in the soil, such as topography, nutrient distribution, and other natural causes.

In this statistical analysis based on remote sensing image analysis, we consider landscapes populated by a dominant Tillandsia landbeckii, neglecting genetic variation between the plant species present in a landscape and ignoring phenotypic differences^68^.

### Theoretical model

An attempt to model hyper-arid ecosystems specifically involving vegetation population of *Tillandsia landbeckii* was proposed in an earlier report^59^. This model is an extension of the Klausmeier-type model involving droplets of water density flowing in the direction of fog coupled with biomass density. In this work, the analysis is limited to one-dimensional dynamics in the direction of fog propagation. We adopt the modelling approach based on the interaction redistribution model^15^ in which the competitive interaction occurs through the roots. However, the *Tillandsia landbeckii* do not have functional roots, so the competition takes place at the level of aerial vegetation in the presence of fog. They absorb water droplets and nutrients from the atmosphere through the trichomes that densely cover their leaves. They can live on the mobile soils of desert dunes, an aspect which is not explicitly considered in our modelling approach. Considering conservative dynamics of soil resources has recently proved useful to further understand arid ecosystems^69,70^, leading to phase separation and their respectively coarsening dynamic over time. Nevertheless, the observation of a well-defined pattern wavelength is consistent with a dissipative mechanism of self-organization captured by the interaction redistribution model. Three modifications are required to model the Tillandsia landbeckii population in relation to the interaction redistribution model^15^: First, we attribute the competitive interaction to the uptake of fog that contains not only water but also nutrients such as minerals. Second, we modify the kernels or influence functions governing the non-local interactions by taking into account the anisotropy due to the fog movement and the sloped nature of the topography. Third, we take into account the nonreciprocal feedback. The spatiotemporal evolution of the biomass density $b(\textbf{r},t)$ obeys the following integrodifferential equation

1 $$\begin{aligned} \partial _t b= b(1-b)M_f[b]-\mu bM_c[b] + D\nabla ^2b, \end{aligned}$$

where *r* and *t* are the spatial and temporal coordinates, $M_{f}$ and $M_{c}$ are functionals of the neighbouring biomass field *b*, and they model the feedback involving vegetation growth enhancement or decay, respectively.

**Figure 3:**
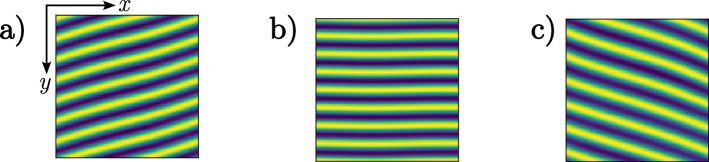
Horizontal and oblique banded patterns obtained by numerical simulations of Eq. (1). (**a**) Oblique banded pattern to the left clockwise (oblique left vegetation pattern), (**b**) horizontal, and (**c**) oblique banded pattern to the right counterclockwise (oblique right), respectively. All numerical patterns are obtained using a noisy initial condition. All patterns are obtained for the same parameters $\mu = 0.98$, $\chi _f=2.1$, $\chi _c=1.2$, $D=0.2$, $x_{0c} =-0.2,\,x_{0f} =0.5, \,y_{0i} =0$, $l_{fx} = l_{fy}=0.9$, $l_{cx}=5$, and $l_{cy}=3.5$.

The nonlocal coupling functions are $M_i[b](\textbf{r})= \exp {[\chi _i\int \phi _i(\mathbf {r,r'})b(\mathbf {r'}) d\mathbf {r'}]}$, where, the subscript *i* stands for facilitative (*f*) and competitive feedback (*c*), and $\chi _i$ measures their intensity; $\phi _i(\mathbf {r,r'})=\exp [-(x-x'-x_{i0})^2/2 l_{ix}^2-(y-y'-y_{i0})^2/2 l_{iy}^2]/2\pi l_{ix} l_{iy}$ are the kernel or influence functions. $D\nabla ^2b$ models the dispersion by seed dispersion which is assumed to be a diffusive process.

The kernel functions are anisotropic due to the environmental conditions, i.e., $\phi _i(\mathbf {r,r'})\ne \phi _i(\mathbf {|r-r'|})$. This means that these functions break the rotational symmetry. However, the kernels are not reciprocal due to the slope and/or the fog, which induces a directionality in the competition for resources, i.e., $\phi _i(\mathbf {r, r'})\ne \phi _i(\mathbf {r', r})$. In the case of water runoff, we assume that the slope is homogeneous, for a driving fog, the direction of the fog flow is, on average, along a uniform wind.

In the following, we approximate the original integrodifferential Eq. (1) describing the dynamics in general with a nonlinear partial differential equation of fourth order. This reduction is valid in the weak gradient limit, where unstable spatial fluctuations have large wavelengths. This means we are looking for conditions close to the critical point associated with nascent bistability where $\mu =1$, $\chi _f-\chi _c = 1$, and $b = 0$. Starting from the interaction-redistribution model Eq. (1) and taking into account anisotropy and nonreciprocity, the deviation *b* from its value at the onset of nascent bistability is shown to obey the following equation ^6^

2 $$\begin{aligned} \partial _t b= -\eta b +\kappa b^2 - b^3/2 + d\nabla ^2b -b\left( \alpha \partial _x +\gamma \partial _x^2 +\partial _x^4\right) b, \end{aligned}$$

where $\eta$ accounts for the balance between linear birth and mortality rate; $\eta$ is positive when the mortality rate is greater than the birth rate. $\kappa$ is the parameter that stands for the quadratic nonlinearity arising due to logistic saturation and kernel effects, often called the cooperativity parameter. $d/\sigma ^{1/2}$ models the seed dispersion. $\alpha$ accounts for the intensity of nonreciprocity. $\gamma$ accounts for the balance between facilitation and competition ranges. The link between the reduced parameters and the coefficients that appear in Eq. (2) are $\kappa = \chi _f-\chi _c-1$, ${\alpha }= (x_{0c}\chi _0 - x_{0f}(1+\chi _0) )/\sigma ^{1/4}$, $\gamma = (\chi _1(l_{cx}^2-l_{fx}^2) )/\sigma ^{1/2},$ with $\chi _0= l_{fx}^2/(l_{cx}^2-l_{fx}^2)$, $\chi _c = \chi _0+\chi _1$ and $\sigma = 3l_{fx}^2 l_{cx}^2$. The space has been rescaled according to $\textbf{r}= \textbf{r}/\sigma ^{1/4}$. The previous equation is valid close to the critical point, that is, $\mu = 1 +\eta$, with $\eta \ll 1$.

**Figure 4:**
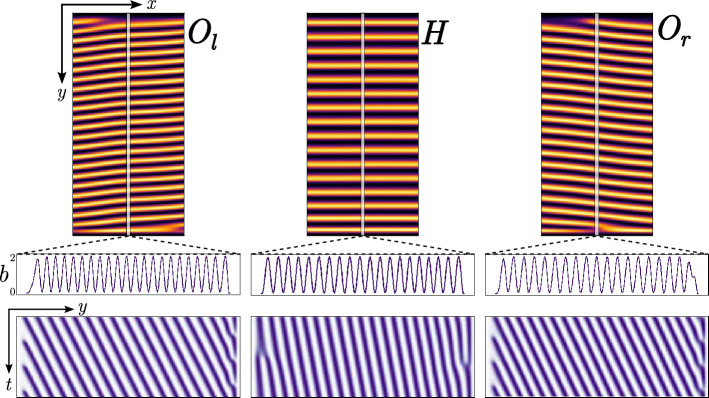
Horizontal and oblique banded patterns obtained by numerical simulations of Eq. (2) with $\eta =-0.02$, $\kappa =0.3$, $d=0.3$, $\alpha =0.27$, and $\gamma = 2$. Columns $O_l$, *H*, and $O_r$ show oblique banded patterns to the left clockwise, horizontal, and oblique banded patterns to the right counterclockwise, respectively. From top to bottom, the first row shows the 2D colour representation of the biomass field *b*(*x*, *y*, *t*). The second row is the biomass profiles *b* along the *y* direction at the central line, and the final row is the spatiotemporal evolution of these profiles, depicting the upward movement or advected patterns.

### Oblique and horizontal banded vegetation patterns: numerical simulations

Numerical simulations of model Eq. (1) are performed using a finite differences code with Runge-Kutta order-4 algorithm and mixed boundary conditions. In the *x*-direction, we use the Dirichlet boundary conditions $b(x=0,y)=b(x=L_x,y)=0$ and in the *y*-direction, we apply periodic boundary conditions, $b(x, y+L_y) = b(x, y)$, with $L_x$ and $L_y$ are the box size in *x* and *y* direction, respectively. The Dirichlet boundary conditions are justified by the fact that the *Tillandsia* bands are localised, since the topography can limit the availability of fog water and restrict plant propagation along the *x*-direction, as shown in Figs. 2 and 7 (see “Materials and methods”). The result of numerical simulations is shown in Fig. 3. To observe horizontal patterns in the spatial monostable region, we consider a noisy initial condition for biomass. We observe different patterns when considering noisy initial conditions in the pattern coexistence region.

The homogeneous solutions of Eq. (2) are identical to the generic interaction and redistribution model derived for isotropic environmental conditions^32^. These solutions are a bare state $b_s=0$ corresponding to landscapes totally devoid of plants and homogeneous plant populations $b_{\pm } = \kappa \pm \sqrt{\kappa ^2 - 2\eta }$. When $\kappa < 0$, only the homogeneous steady state $b_{s+}$, defines the biomass density, for $\eta < 0$. It decreases monotonically with $\eta$ and disappears at $\eta = 0$. When $\kappa > 0$, the homogeneous branch of the solution extends to the tipping point $b_l = \kappa /2$ and $\eta _l= \kappa ^2/4$. In the interval $0< \eta < \eta _l$, biomass density exhibits bistable behaviour between the homogeneous branches of solutions $b_{s\pm }$ and $b_s=0$.

We fix all parameters, vary the aridity parameter $\eta$, and focus our analysis on the bistable regime $\kappa > 0$. The reduced Eq. (2) is integrated numerically in a rectangular-shaped domain subjected, as in the case of Eq. (1), to Dirichlet and periodic boundary conditions for the $\textbf{x}$ and $\textbf{y}$ direction, respectively. The initial condition is chosen to be random for the phytomass density. The results are summarised in Fig. 4. The spatiotemporal behaviour undergoes banded patterns with a wavevector oriented in the $\textbf{x}$ direction. When $\alpha \ne 0$, banded vegetation patterns are moving due to nonreciprocal facilitative and competitive feedback. As in the case of model Eq. (1), two types of vegetation patterns are generated numerically: oblique and horizontal propagative bands, as shown in Fig. 4. Therefore, the non-local model Eq. (1) and its respective local approximation Eq. (2) account for the coexistence of propagative band vegetation patterns. This behaviour resembles what is observed in *Tillandsia landbeckii* vegetation patterns, where for a single patch, different angles with the fog propagation direction are well defined (cf. Fig. 2). However, the patterns observed in northern Chile exhibit spatial irregularities and dislocation defects.

### Oblique and horizontal banded vegetation patterns: linear and nonlinear analysis

**Figure 5:**
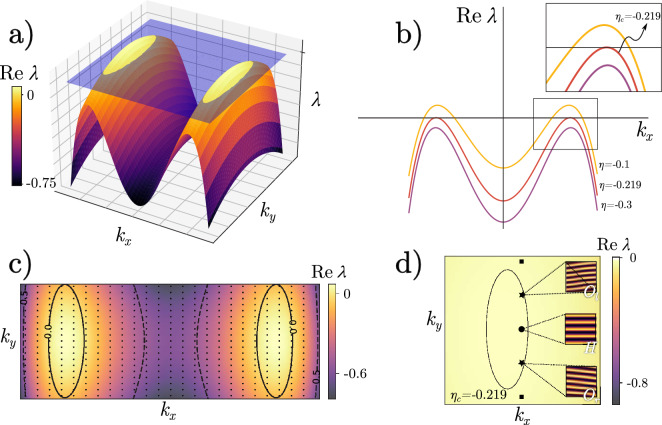
Linear analysis close to the onset pattern formation. Panel (**a**) represents the Fourier space for $\text {Re }~\lambda (k)$, where the blue plane indicates $\lambda =0$. The condition for pattern formation is depicted in (**b**) as a projection; as $\eta$ increases towards a critical value $\eta _c$, the curve surpasses the $\{k_x, k_y\}$ plane, allowing a band of unstable modes to appear. This region is shown in (**c**), where the discrete unstable modes, represented by dots, are within the solid line, while those outside satisfies $\text {Re }~\lambda (k)<0$. For $\eta =-0.219$, there are three unstable modes, as shown in (**d**), two obliques $O_l$ and $O_r$, and the horizontal one *H*.

Nonlinear study through normal form analysis is required to understand horizontal and oblique banded vegetation patterns. To do this, let us first linearise the Eq. (2) of the homogeneous cover $b_{s+}$, and consider a spatial perturbation of the form $b=b_{s+}+b_o e^{\lambda t+\textbf{k r}}$ where $\textbf{k}=(k_x,k_y)$, **r**=(x,y) and $b_0\ll 1$, which yields a characteristic equation for the growth rate $\lambda$ as a function of wavenumber $\textbf{k}$. The threshold associated with the pattern forming instability requires two conditions: $\partial _\textbf{k} \text {Re }\lambda (\textbf{k}) |\mathbf {k_c} =0$ and $\text {Re }\lambda (\mathbf {k_c})=0$. We fix all parameters, and we consider the aridity $\eta$ as a control parameter. These two conditions determine the critical wavenumber $\mathbf {k_c}$ and the critical aridity $\eta _c$ . The summary of the linear analysis is shown in Fig. 5. Above the critical threshold, i.e., $\eta >\eta _c$ then $\text {Re} \lambda >0$, two ellipsoids of unstable spatial modes appear in Fourier space as shown in Fig. 5a,b. The Dirichlet boundary conditions along the *x* direction impose that unstable modes are discrete, i.e., $k_y = 2 \pi n/L_y$ and $k_x = \pi m/L_x$ for integers *n* and *m*. As the aridity parameter increases, the spatial discrete unstable oblique modes appear, as depicted in Fig. 5c,d. These figures reveal that the first pattern to appear for increased mortality corresponds to bands with a wavenumber parallel to the $\textbf{x}$ direction; then, a symmetric pair of oblique banded patterns appear for enough aridity, see Fig. 5d. The linear analysis predicts that any superposition of these patterns should be a solution; however, this is not observed in numerical simulations or field observations. Of course, nonlinear saturation and interaction of these Fourier modes play an essential role in selecting the well-defined banded patterns as observed in nature.

To determine the solutions, such as horizontal and oblique banded patterns that have been numerically obtained and observed by remote sensing image analysis, we conduct a weakly nonlinear analysis. The solution of Eq. (2) can be approximated by $b=b_{s+}+ A(\textbf{r},t)e^{ik_cx+i\Omega _c t}+c.c.$ where *c*.*c*. accounts for complex conjugate. The complex amplitude $A(\textbf{r},t)$ of the banded vegetation pattern near the threshold associated with pattern forming process obeys a Ginzburg-Landau type of equation^6^

3 $$\begin{aligned} \partial _t A= \hat{\mu } A- (1+i\beta )|A|^2A +\nabla ^2A-\hat{\alpha }\partial _xA, \end{aligned}$$

$\hat{\mu }$ is proportional to the critical mode growth rate, $\beta$ is a nonlinear dispersion, and $\hat{\alpha }$ is proportional to the group velocity of the pattern, which arises from relation dispersion $\text {Im} (\lambda (\textbf{k}))$, specifically $\hat{\alpha } = \partial \text {Im}(\lambda )/\partial k_x$ and corresponds to the speed of propagation of the pattern bands. See reference^6^ for a detailed derivation and parameter expressions. If the reflection symmetry is not broken, the model equation will be invariant with respect to the transformation $\textbf{r} \rightarrow \mathbf {-r}$ and rotation of coordinates, then, the banded pattern will be motionless^14^. However, if the interaction is nonreciprocal, the reflection symmetry is broken, as in the case of Ginzburg-Landau Eq. (3). Therefore, the banded vegetation pattern exhibits a global motion, in this case towards the incoming fog.

The solution of the horizontal banded pattern is $A\equiv H= H_0e^{i\phi _0}$, with $H_0$ the pattern amplitude and $\phi _0$ an additive phase. The solution of oblique banded patterns is $A(y)\equiv O_{l,r} e^{i(\phi _0 \pm \Delta k y) }$, with $O_{l,r}$ representing the oblique patterns amplitude towards the left and right flanks, respectively. The horizontal and oblique patterns are described by the wavevector $\mathbf {k_H} = (k_c, 0)$, and $\mathbf {k_O} = (k_c, \Delta k)$, respectively. Note that $\Delta k$ is of order $\sqrt{ \hat{\mu }}$.

To calculate the solutions emerging from the pattern-forming instability, we use a standard nonlinear analysis based on a truncated Fourier mode expansion of the field $A(\textbf{r},t)$. This analysis allows us to determine both horizontal and oblique banded patterns, and assess their stability. Then, solutions of the Ginzburg-Landau Eq. (3) can be approximated by a superposition of horizontal and oblique modes as

4 $$\begin{aligned} A= H(t) + O_l(t)e^{i\Delta k y} + O_r(t)e^{-i\Delta k y}. \end{aligned}$$

By replacing the ansatz (4) in Eq. (3), collecting the coefficients proportional to $\{1, e^{i\Delta k y}, e^{-i\Delta k y} \}$, and neglecting higher modes, we obtain the following amplitude equations

5 $$\begin{aligned} \partial _t H&= \hat{\mu } H -(1+i\beta )H\left( |H|^2 + 2\left( |O_l|^2 + |O_r|^2 \right) \right) -2(1+i\beta )O_lO_r\bar{H}, \\ \partial _t O_l&= (\hat{\mu } - \Delta k^2) O_l -(1+i\beta )O_l\left( 2|H|^2 + |O_l|^2 + 2|O_r|^2 \right) -(1+i\beta )H^2\bar{O_r}, \\ \partial _t O_r&= (\hat{\mu } - \Delta k^2) O_r -(1+i\beta )O_r( 2\left( |H|^2 + |O_l|^2) + |O_r|^2 \right) -(1+i\beta )H^2\bar{O_l}. \end{aligned}$$

**Figure 6:**
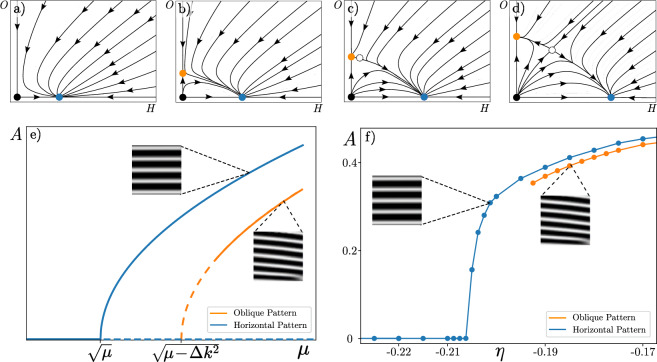
Bifurcation diagram of horizontal and oblique banded vegetation patterns. The top row shows projections of the phase portrait for the oblique ( *O*) and horizontal ( *H*) banded patterns amplitudes. The uniform state corresponds to the origin of the phase portrait, which is represented by a black dot. The first pattern to arise is the horizontal one, depicted as the blue dot, emerging from the homogeneous state. Increasing the bifurcation parameter, $\hat{\mu }$, the oblique pattern (orange dot) emerges from the homogeneous state, as observed in (**b**). Note that, at first, this pattern is unstable, and through the appearance of the mixed pattern (white dot), the pattern becomes stable, as shown in (**c**). As a result, (**d**) illustrates the full phase space for the oblique and horizontal banded pattern amplitudes. This dynamic is represented in panel (**e**) as a bifurcation diagram. Moving forward $\hat{\mu }$, first arises the horizontal pattern in a supercritical way (blue curve), then the oblique pattern emerges supercritically. The solid and dashed lines account for stable and unstable states. The panel (**f**), is the bifurcation diagram obtained by numerical simulation of Eq. (2) by $\kappa =0.3$, $d=0.3$, $\alpha =0.5$, and $\gamma = 2$, showing the same behaviour of panel (**e**).

The set of Eqs. (5) describes the dynamics of banded patterns, that is, given an initial condition composed of horizontal and oblique stripes, the amplitude of each one is determined in the future by integrating in time Eqs. (5). Then, we are interested in the different equilibria that Eqs. (5) exhibit, as well as their stability. One can see that for $\hat{\mu }<0$ the only equilibrium is $(H, O_l, O_r)=(0, 0, 0)$, this is because the system is in a non-pattern forming regime and the homogeneous solution is the stable one. When increasing the bifurcation parameter $\hat{\mu }$ such that $\hat{\mu }>0$, first appears the horizontal band pattern from the uniform state $b_{s+}$ (see Fig. 6a), and after a second threshold, the unstable oblique patterns emerge from the homogeneous solution (cf. Fig. 6b); this behaviour is depicted in phase space portraits projection as shown in the bottom panels Fig. 6. Further varying the control parameter, the oblique pattern is stabilised through the emergence of an unstable mixed mode from it (see Fig. 6c). Hence, the oblique and horizontal banded patterns coexist. Due to the dynamics of modes, the analytical bifurcation diagram depicted in Fig. 6e is obtained. This figure shows a parameter range for which only horizontal banded patterns are possible in finite systems but also a region of coexistence for both patterns. Moreover, through direct simulation of Eq. (2), we obtain an analogous bifurcation diagram depicted in Fig. 6f, describing the same phenomenon using a bifurcation parameter $\eta$, one zone with only the existence of a horizontal banded pattern and another with both horizontal and oblique patterns as stable solutions. The coexistence of horizontal and oblique banded patterns is in agreement with the remote observations as shown in Fig 2h.

## Conclusions

We studied the vegetation patterns of populations of Tillandsia landbeckii. This plant survives in the hyperarid landscapes of the Atacama Desert in Chile and Peru. This plant has no functional roots and lives on slopes in extreme environmental conditions. Tillandsia landbeckii survives thanks to the fog the plants trap with their dense aerial structure. We used remote sensing image analysis and mathematical modelling to address the spatiotemporal evolution of banded vegetation patterns and their orientation.

Using remote sensing data analysis, we constructed histograms showing the angular deviation between the prevailing wind direction in which the fog flows and the banded vegetation pattern. We identified three main directions, one perpendicular to the prevailing wind and two inclined at about 7^∘^, coexisting in the same area. We have shown that the preferred direction of banded vegetation patterns is due to fog flow rather than topography.

Using the interaction-redistribution model with nonreciprocal effects mediated by non-local facilitative and competitive interactions between plants, we showed evidence of oblique banded vegetation patterns. The origin of the nonreciprocal interaction lies in the flow of fog along the prevailing wind. This effect influences seed dispersal and can favour plant reproduction in the wind direction. Starting from the full integrodifferential model, we have reduced it to a simpler model, the partial-differential model. This reduction is valid in the weak gradient limit, where a large-wavelength pattern formation occurs. We have provided evidence of horizontal and oblique vegetation patterns through numerical simulations of both models. From the simplified model, we have performed a linear stability analysis, which shows that the spectrum of unstable Fourier modes is discrete due to the Dirichlet boundary conditions along the fog direction. A nonlinear analysis is also performed, which allows for establishing a normal form associated with horizontal and oblique banded vegetation patterns. This analysis allows us to obtain a bifurcation diagram and access the stability of the three nonlinear solutions.

Our theoretical description predicts a scenario where coexistence exists far from the transition between the vegetation pattern and uniform vegetation cover state (see Fig. 6). Therefore, the observation of both pattern orientations, meaning bistability, is a manifestation of environmental stress on the system. The presence of different orientation domains within the same vegetation patch generates defects. The dynamics of these defects in vegetation self-organization and their ecological consequences remain poorly understood. Studies in this direction are in progress.

**Figure 7:**
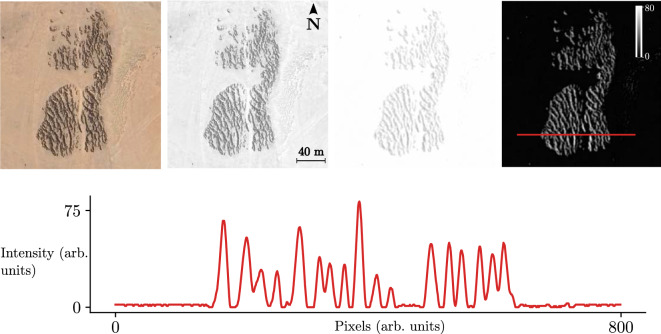
Tillandasia landbeckii vegetation patterns. From left to right of the top panels, the first panel is a satellite image from *Tillandsia* patches. The next one corresponds to the same picture but in greyscale. A Gaussian filter was used to smooth the pattern for the third image, and the background was removed. As a result, we obtain the last panel, but the greyscale is inverted. The bottom panel shows a horizontal spatial profile of the *Tillandsia landbeckii* pattern highlighted in the red line on the right top panel; the peaks account for biomass, and the minimums are bare soil.

By employing a straightforward modelling approach and analysing remotely sensed images, we were able to discern oblique and horizontal bands with overlapping domains of stability. This was achieved by studying the relationship between the orientation of *Tillandsia landbeckii* populations and the topographical gradient and dominant wind direction. In particular, our modelling approach, supported by field observations, suggests that topography does not play a role in the stabilisation of oblique banded vegetation patterns. Note that most spatio-temporal modelling approaches for dryland ecosystems use periodic boundary conditions. Here we explore the use of more realistic boundary conditions that capture the spatial confinement of vegetation patterns and show that this has an effect on the threshold that dictates the appearance of oblique vegetation stripes, which is influenced by the size of spatial confinement (cf. Fig. 7).

## Materials and methods

### Boundary conditions

To justify the Dirichlet boundary conditions that we use along the fog direction, we consider the satellite image as shown in Fig. 7a. The landscape is formed by a dominant species of Tillandasia landbeckii vegetation patterns. This pattern is confined in space, forming a localised vegetation pattern.

### Image acquisition and processing

Satellite images are obtained thanks to the Google Earth software ^67^. They are processed using the FIJI (ImageJ) open software^71^. As illustrated in Fig. 7, the images are first transformed to grayscale (for example, averaging the RGB spectral bands). A Gaussian filter allows for the smoothing of imperfections due to the topography and plant roughness; mathematically, it corresponds to the convolution of the image with a Gaussian kernel of a given pixel width, in this case 1 pixel. Finally, the background is removed with the rolling ball algorithm built-in FIJI^71^, with a radius of 20 pixels.

For obtaining the banded pattern orientations, the plugin OrientationJ is used^72–74^. The algorithm of OrientationJ is based on the use of the structure tensor. The structure tensor is a matrix obtained from the computation of the gradient of the image under analysis. The image of the orientation of the pattern in radians is then obtained.

### Slope and wind data acquisition

Slope information is obtained by drawing different paths in Google Earth^67^, which gives information on the altitude along the path. Then, an altitude field is reconstructed employing the QGIS software tools.

Wind information is extracted from the public database^66^. The wind direction information is given in intervals of $30^\circ$, and the dominant direction for the speed angular distribution corresponds to $30^\circ$ counterclockwise from the East.

### Correlation between pattern, slope and wind angles

Considering that every angle $\gamma _i$, corresponds to a pixel in the image, then every pixel can be associated with a vector, such as for a pixel p, $p = \cos (\gamma _i ){\textbf {x}} + \sin (\gamma _i){\textbf {y}}$. Then, the angular difference between two vectors is the inner product of both, so $\langle \gamma _i, \gamma _{\textit{pattern}} \rangle = \cos ( \gamma _i- \gamma _{\textit{pattern}})$. The angle used to compute the correlation is perpendicular to $\gamma _{\textit{pattern}}$. In this way, the calculation of the correlation measures how aligned the pattern propagation direction is with respect to the wind or slope gradient rather than how aligned the stripes of the pattern are with respect to the other variables mentioned before. The analysis was done in several vegetation patches, ten to be precise, in Coquimbo region, Chile

### Supplementary Information

Supplementary Information.

## Data Availability

The datasets analysed during the study are openly available in supplementary material within this article.
