# Supplementary figures and images for: Nonreciprocal feedback induces migrating oblique and horizontal banded vegetation patterns in hyperarid landscapes
Source: Sci Rep. 2024 Jun 25;14:14635. doi: 10.1038/s41598-024-63820-3 (PMC11199605; doi:10.1038/s41598-024-63820-3)

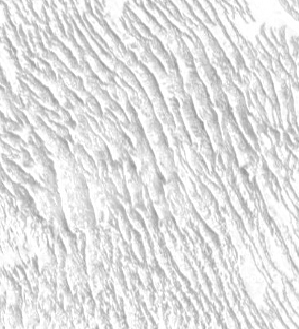

Supplement: Supplementary file 1 — Supplementary Information. [file 41598_2024_63820_MOESM1_ESM.zip › Data/Til/Pattern.tif]

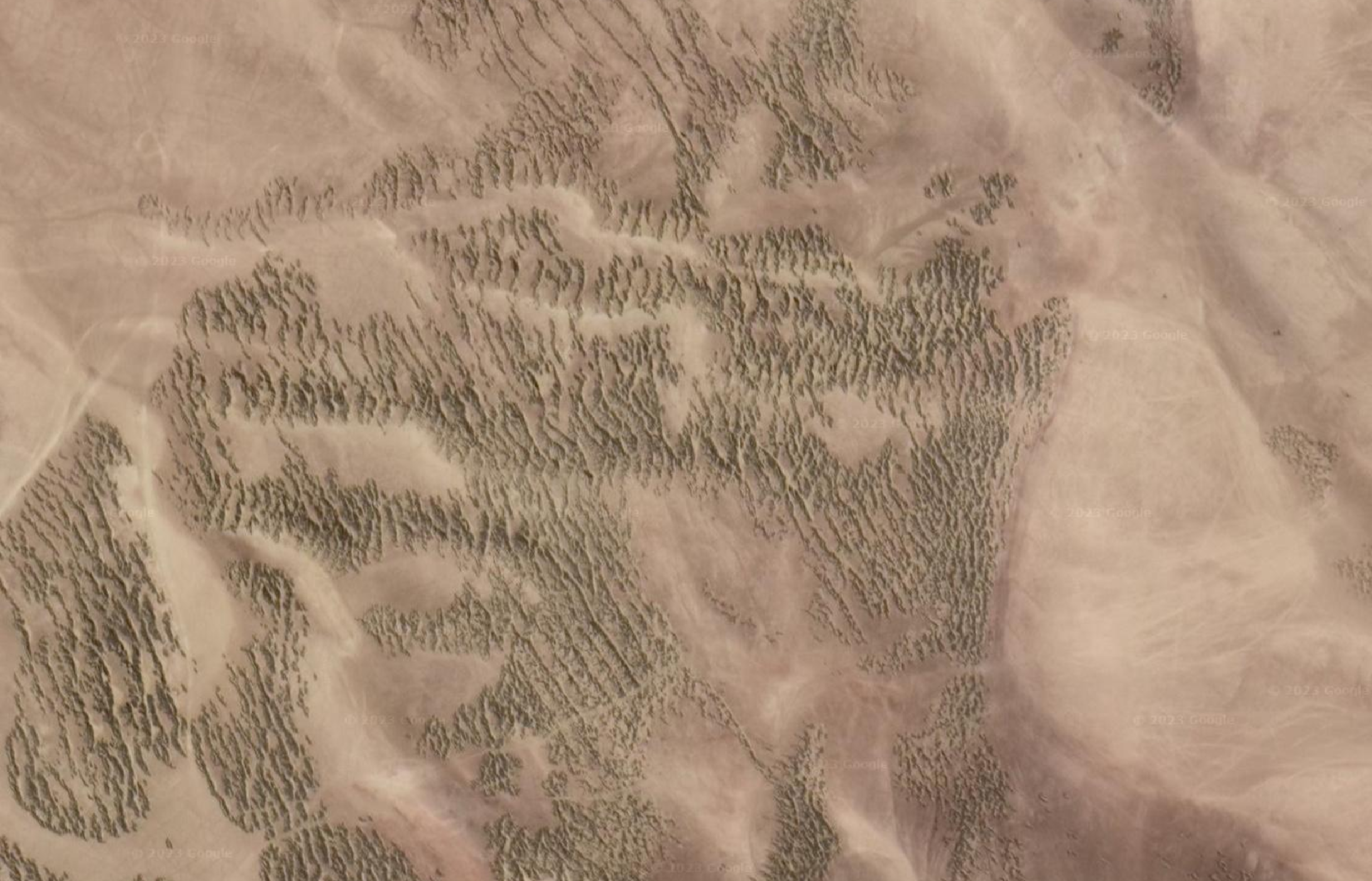

Supplement: Supplementary file 1 — Supplementary Information. [file 41598_2024_63820_MOESM1_ESM.zip › Data/Tilandsias/Til10/Pattern.tif]

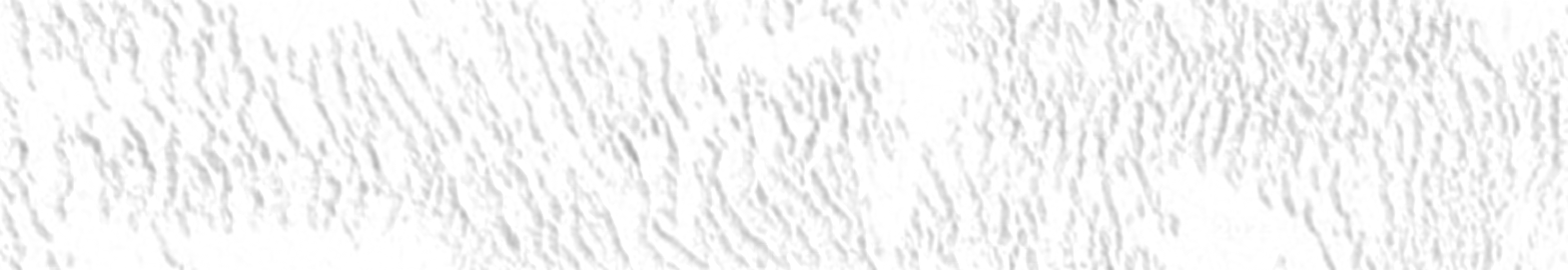

Supplement: Supplementary file 1 — Supplementary Information. [file 41598_2024_63820_MOESM1_ESM.zip › Data/Tilandsias/Til10/Filtered.tif]

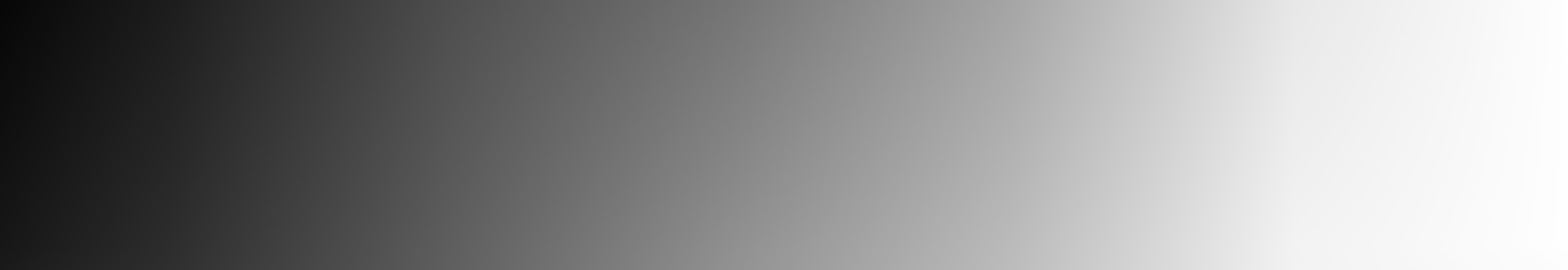

Supplement: Supplementary file 1 — Supplementary Information. [file 41598_2024_63820_MOESM1_ESM.zip › Data/Tilandsias/Til10/ProfileCrop.tif]

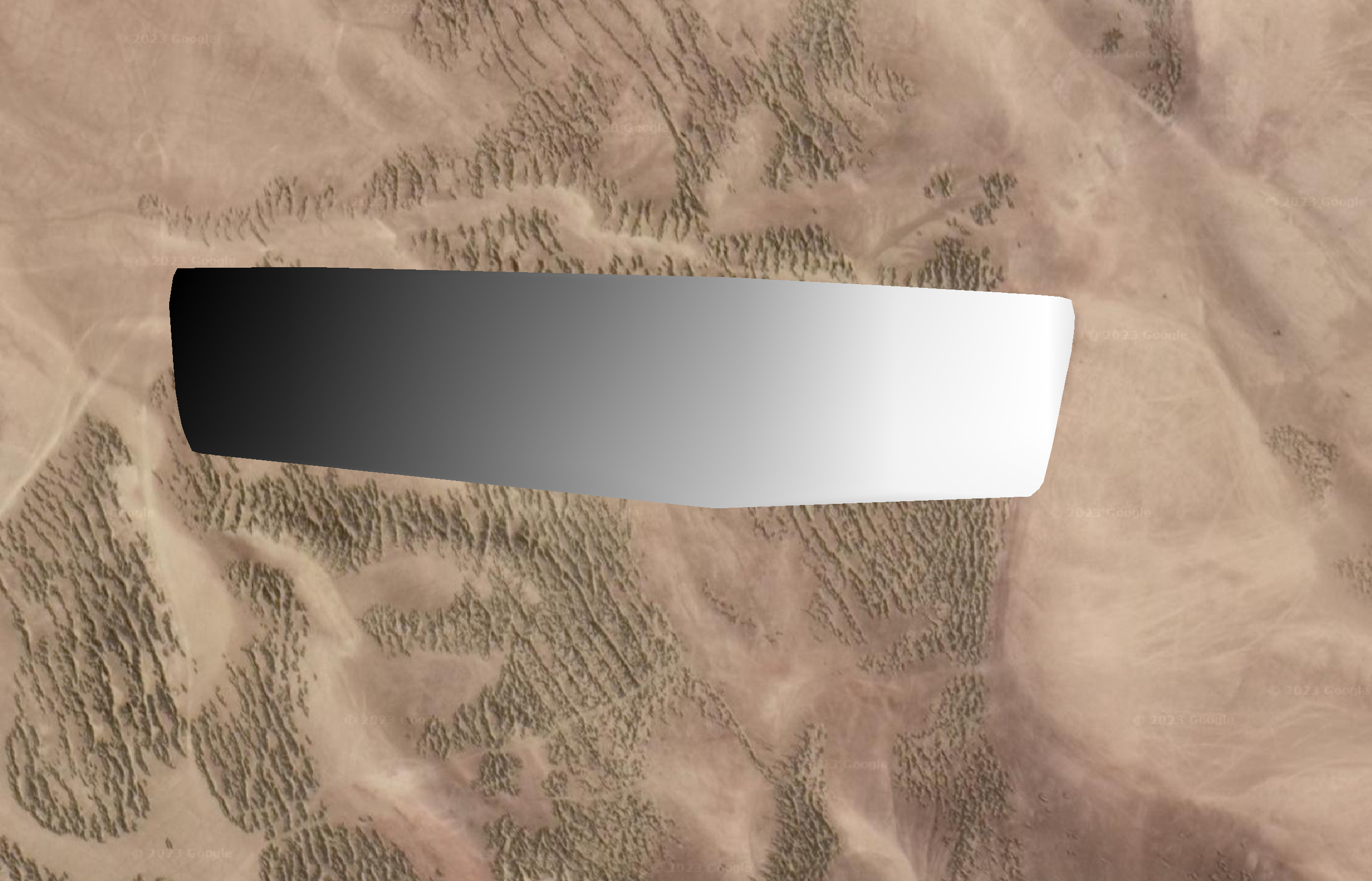

Supplement: Supplementary file 1 — Supplementary Information. [file 41598_2024_63820_MOESM1_ESM.zip › Data/Tilandsias/Til10/Profile.tif]

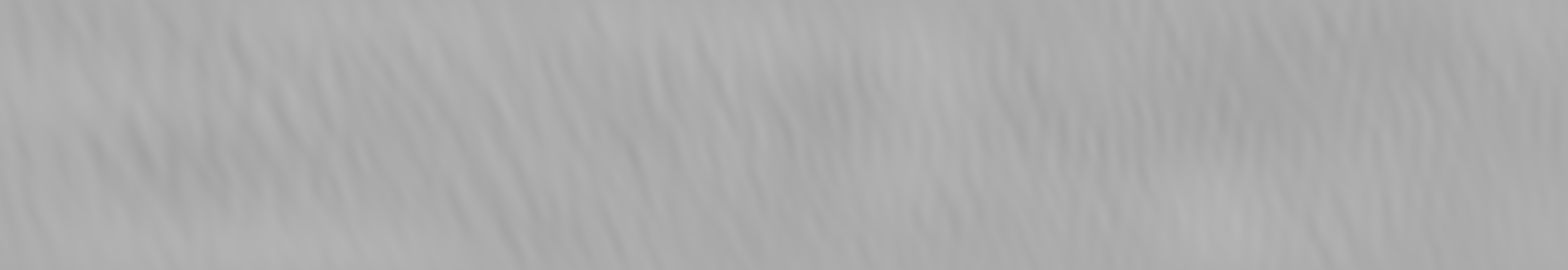

Supplement: Supplementary file 1 — Supplementary Information. [file 41598_2024_63820_MOESM1_ESM.zip › Data/Tilandsias/Til10/InvFFT.tif]

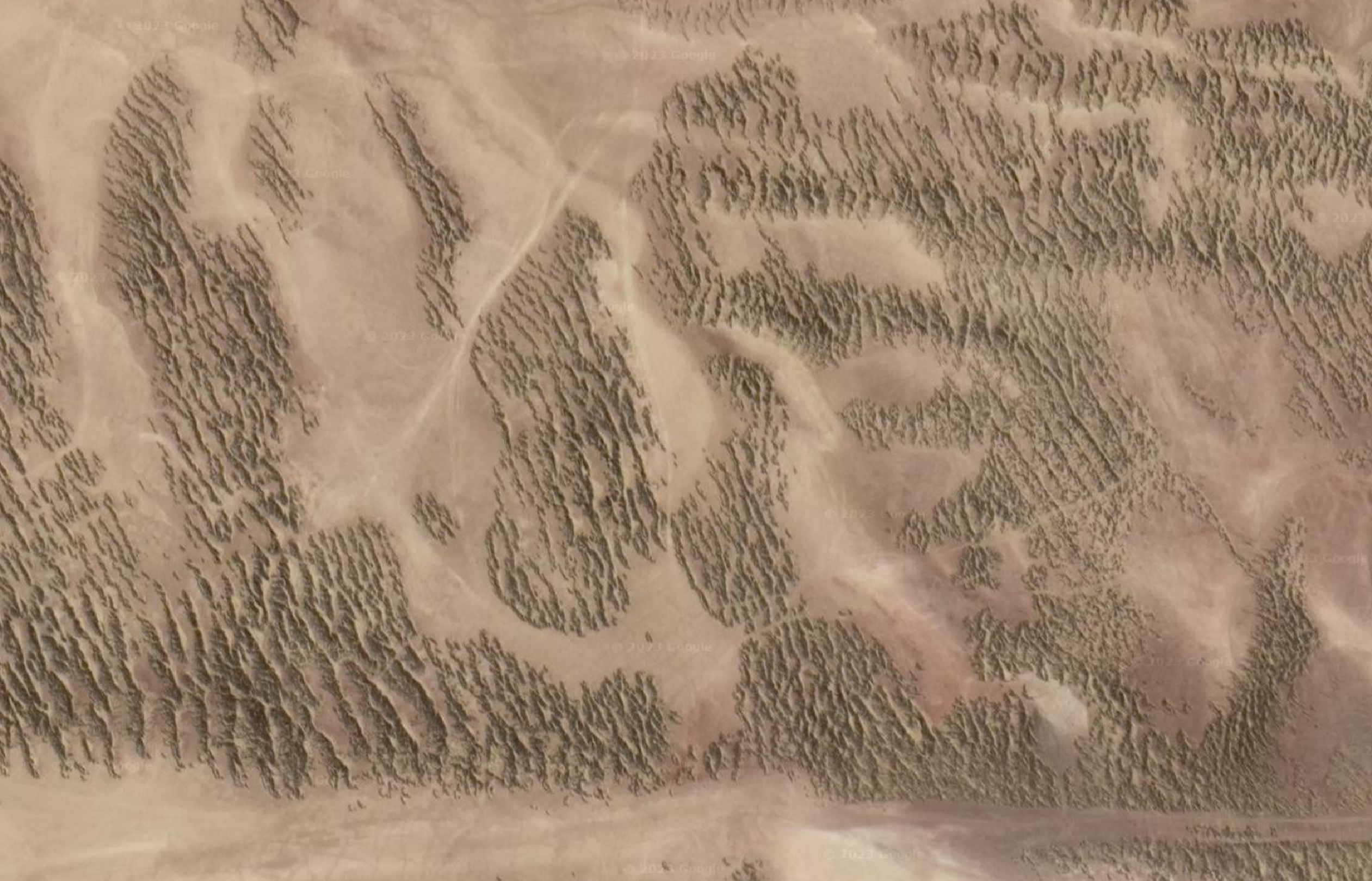

Supplement: Supplementary file 1 — Supplementary Information. [file 41598_2024_63820_MOESM1_ESM.zip › Data/Tilandsias/Til4/Pattern.tif]

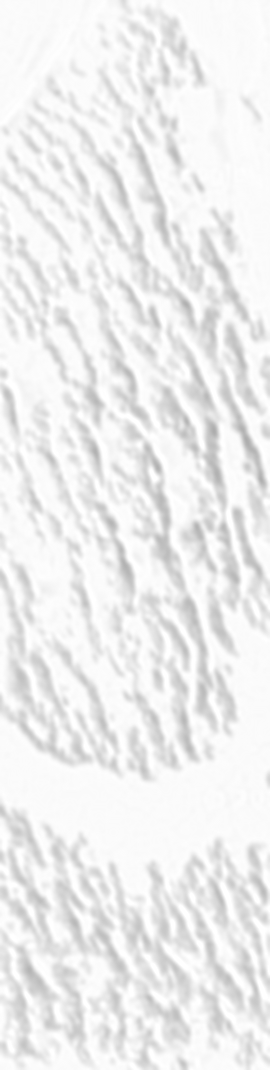

Supplement: Supplementary file 1 — Supplementary Information. [file 41598_2024_63820_MOESM1_ESM.zip › Data/Tilandsias/Til4/Filtered.tif]

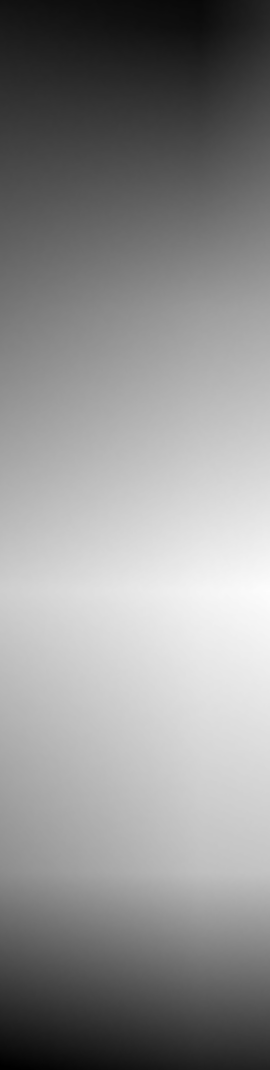

Supplement: Supplementary file 1 — Supplementary Information. [file 41598_2024_63820_MOESM1_ESM.zip › Data/Tilandsias/Til4/ProfileCrop.tif]

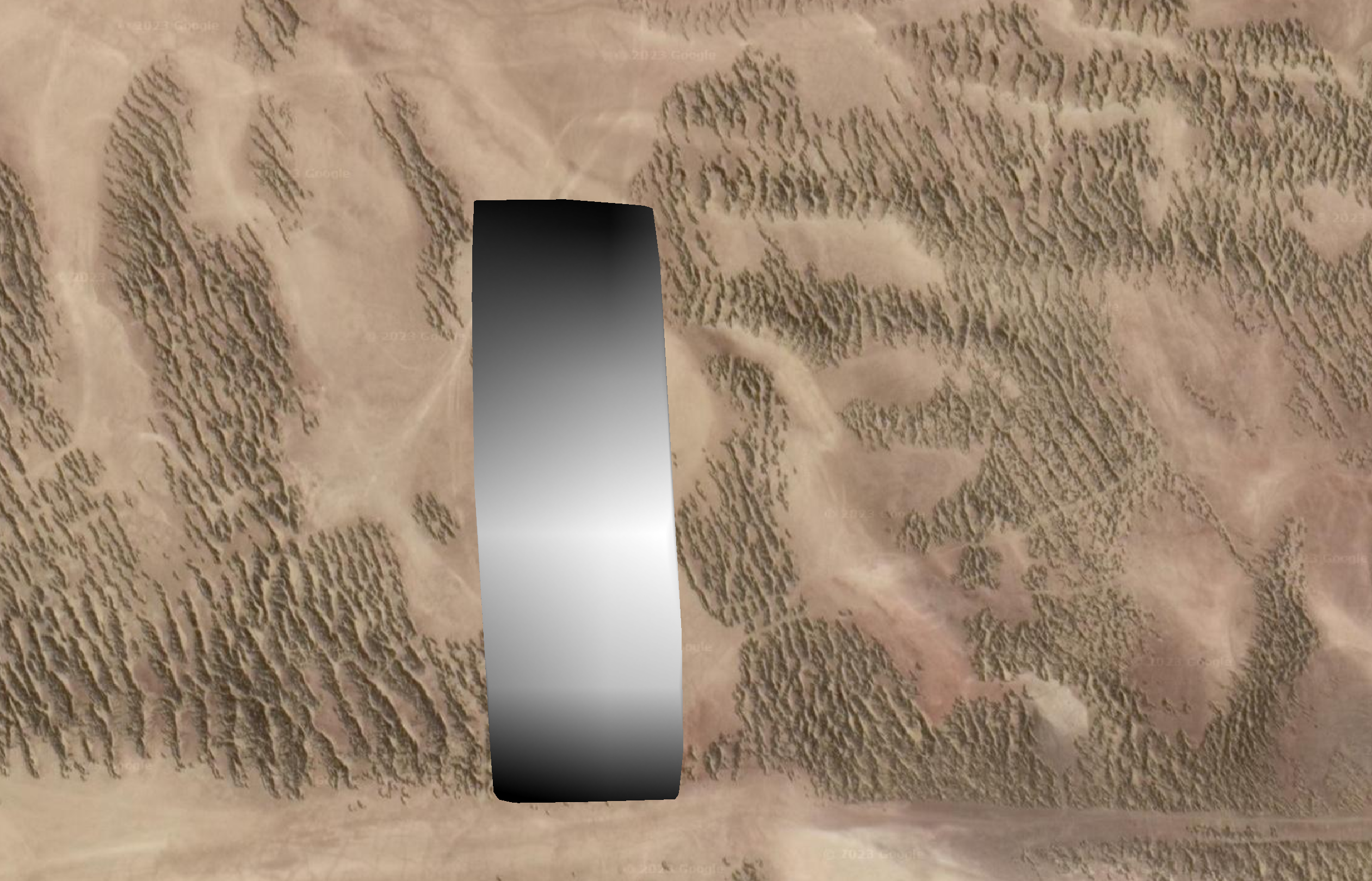

Supplement: Supplementary file 1 — Supplementary Information. [file 41598_2024_63820_MOESM1_ESM.zip › Data/Tilandsias/Til4/Profile.tif]

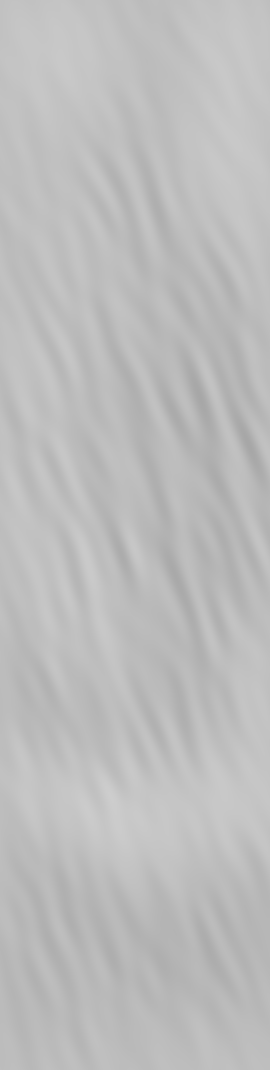

Supplement: Supplementary file 1 — Supplementary Information. [file 41598_2024_63820_MOESM1_ESM.zip › Data/Tilandsias/Til4/InvFFT.tif]

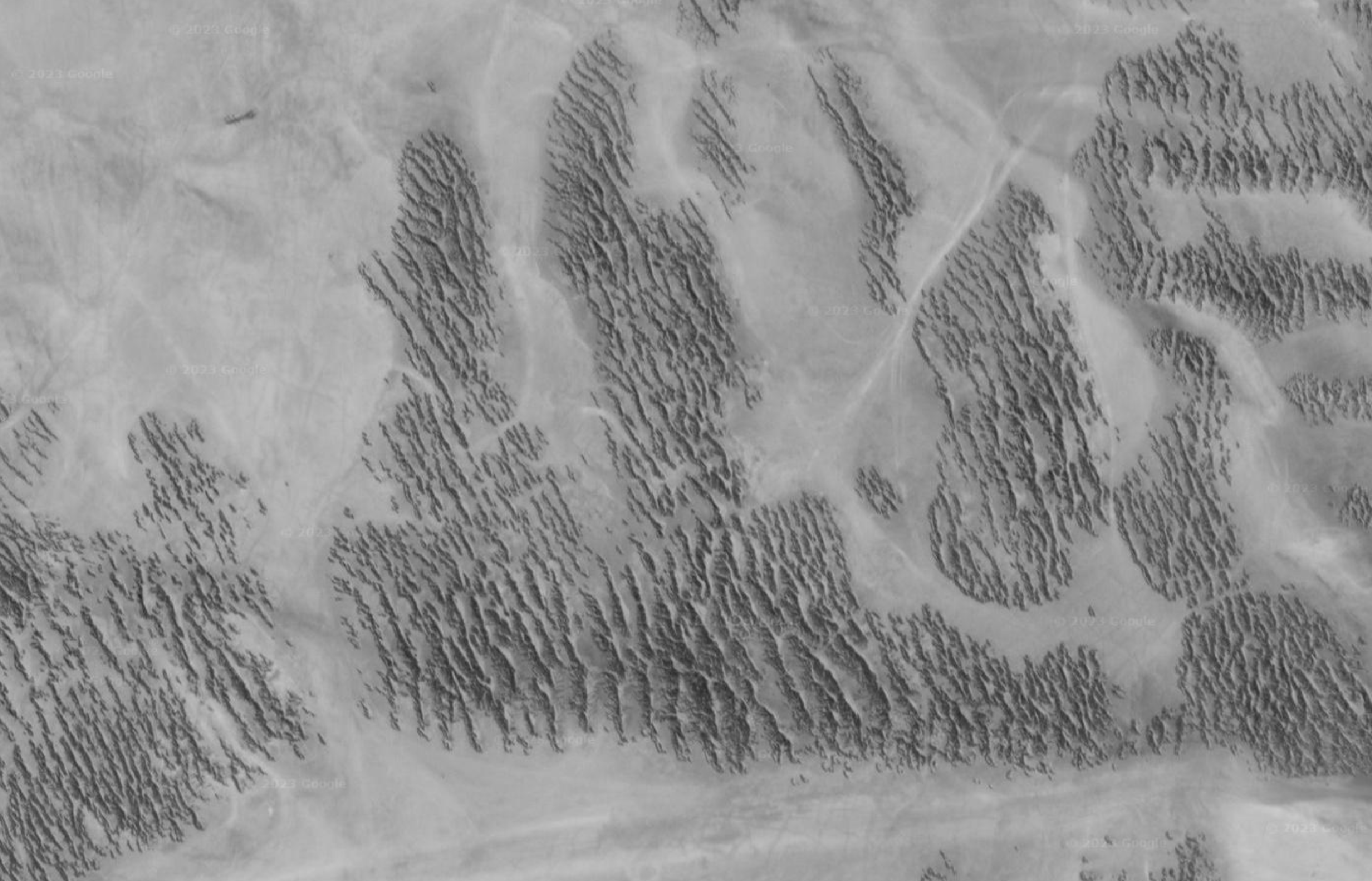

Supplement: Supplementary file 1 — Supplementary Information. [file 41598_2024_63820_MOESM1_ESM.zip › Data/Tilandsias/Til3/Pattern.tif]

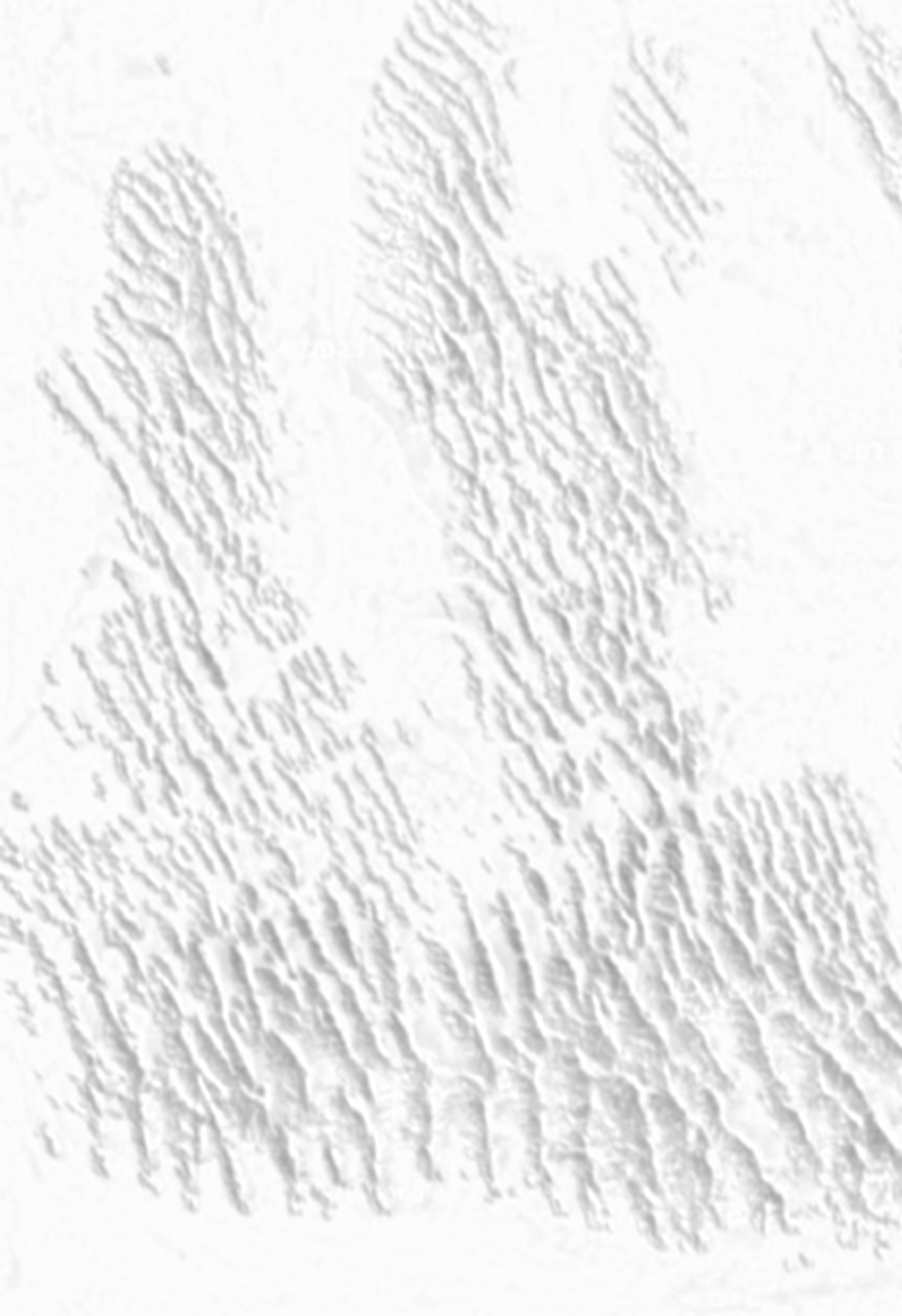

Supplement: Supplementary file 1 — Supplementary Information. [file 41598_2024_63820_MOESM1_ESM.zip › Data/Tilandsias/Til3/Filtered.tif]

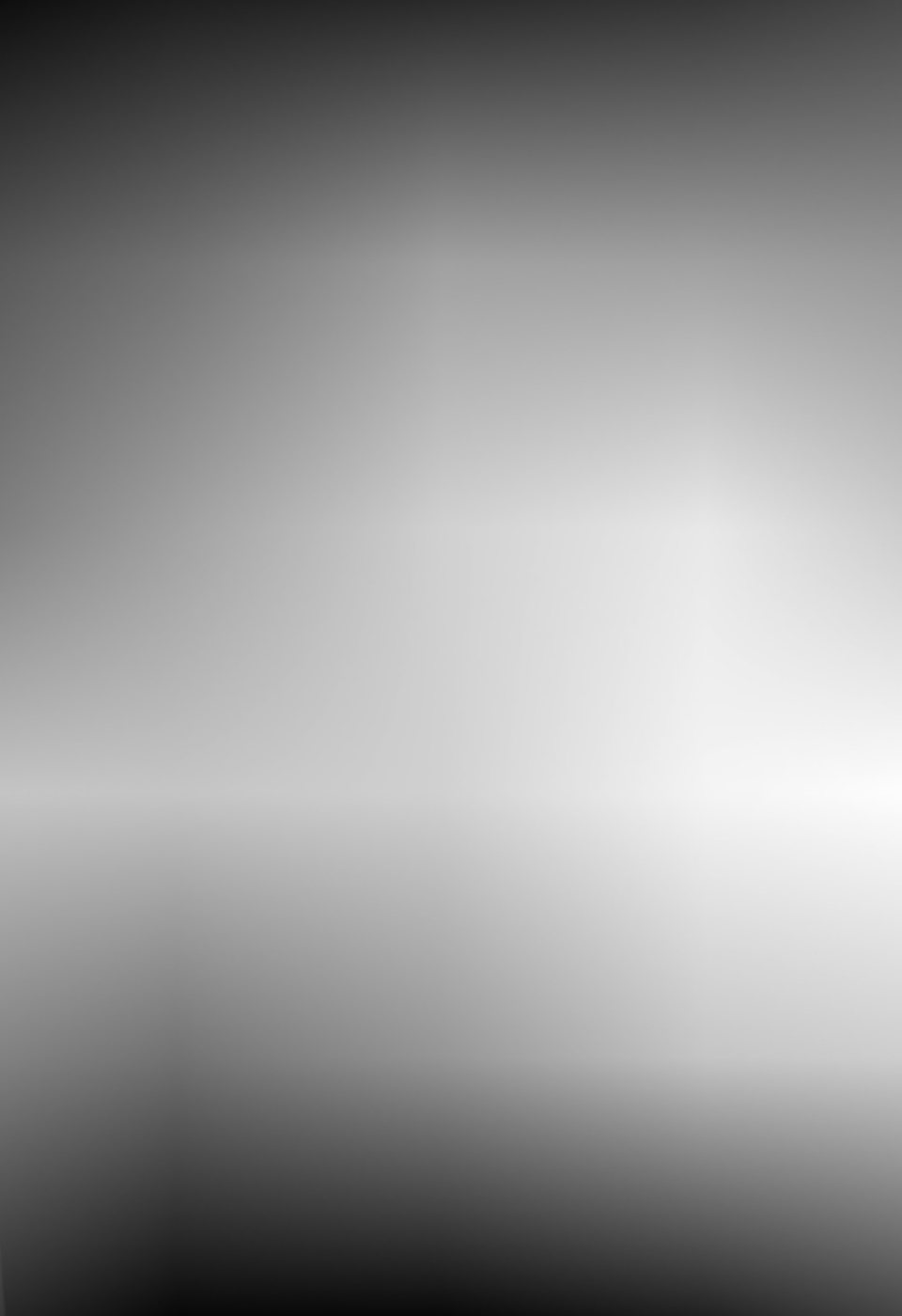

Supplement: Supplementary file 1 — Supplementary Information. [file 41598_2024_63820_MOESM1_ESM.zip › Data/Tilandsias/Til3/ProfileCrop.tif]

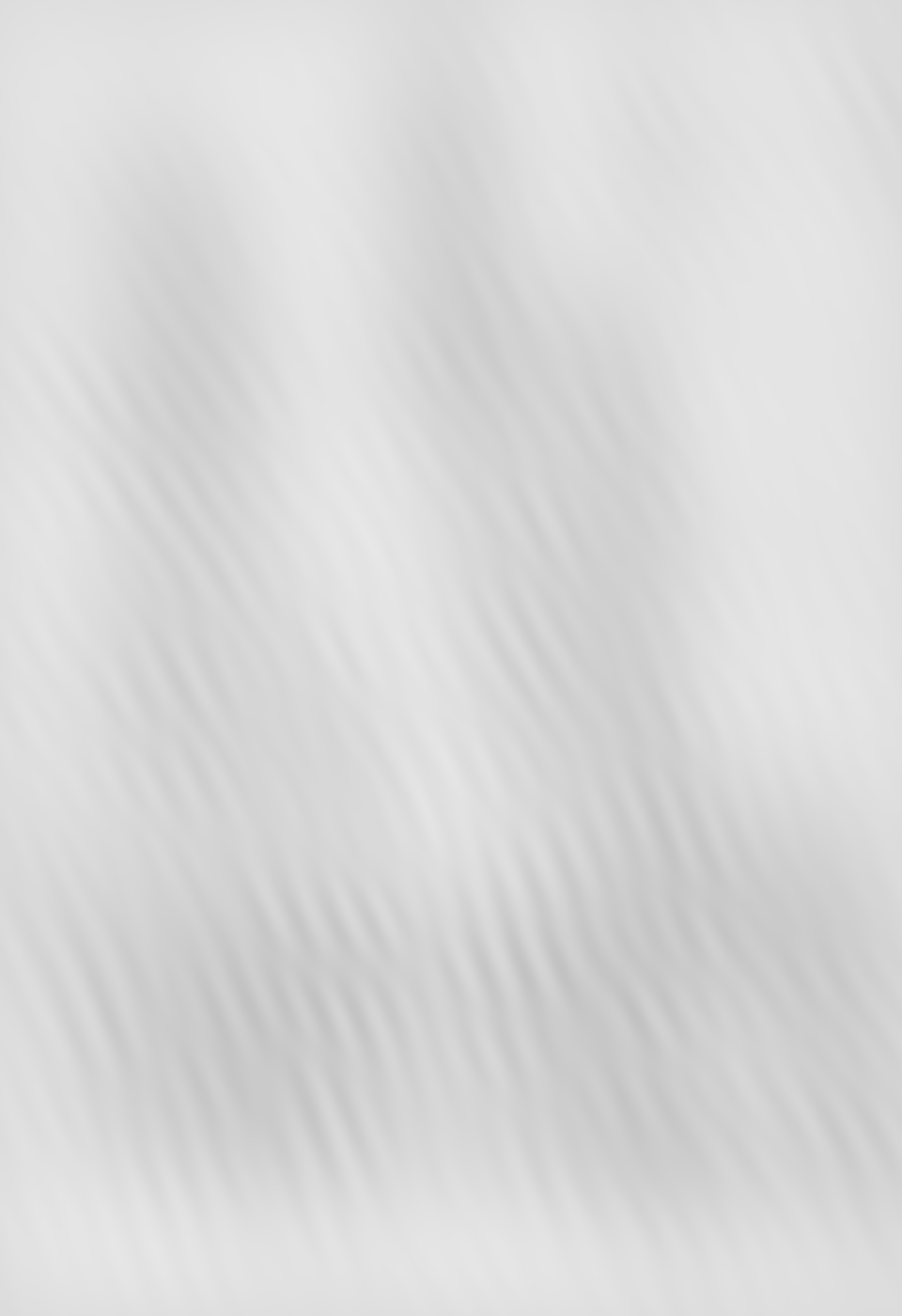

Supplement: Supplementary file 1 — Supplementary Information. [file 41598_2024_63820_MOESM1_ESM.zip › Data/Tilandsias/Til3/InvFFT.tif]

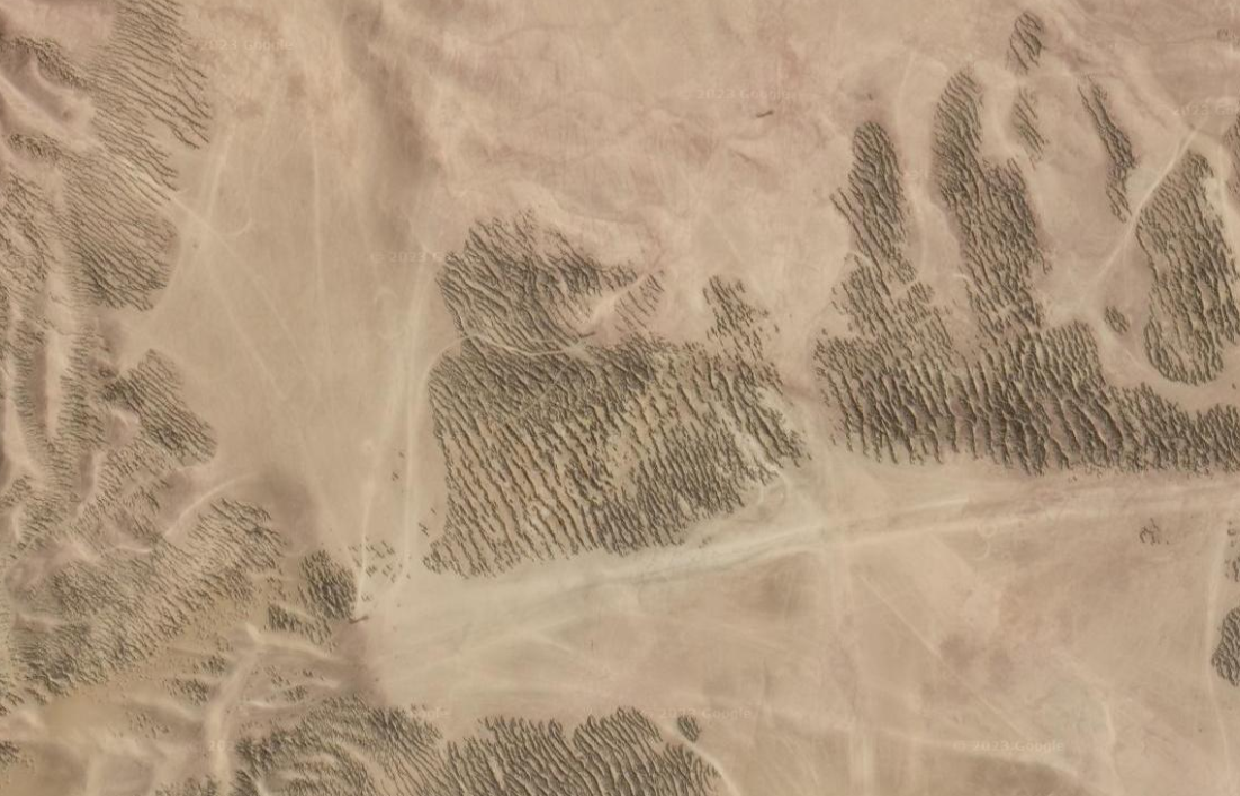

Supplement: Supplementary file 1 — Supplementary Information. [file 41598_2024_63820_MOESM1_ESM.zip › Data/Tilandsias/Til2/Pattern.tif]

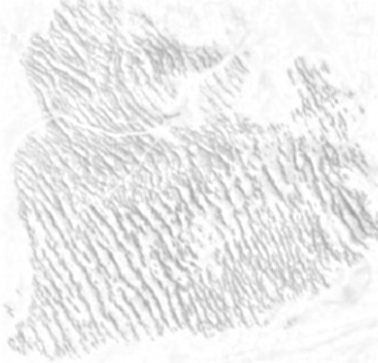

Supplement: Supplementary file 1 — Supplementary Information. [file 41598_2024_63820_MOESM1_ESM.zip › Data/Tilandsias/Til2/Filtered.tif]

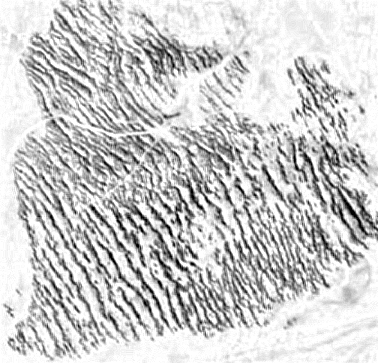

Supplement: Supplementary file 1 — Supplementary Information. [file 41598_2024_63820_MOESM1_ESM.zip › Data/Tilandsias/Til2/Filtered_paper.tif]

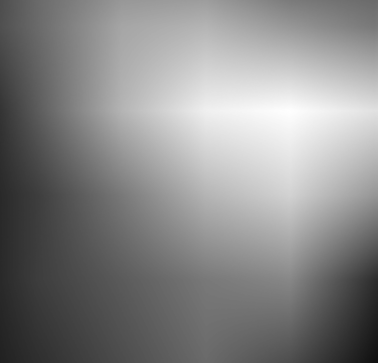

Supplement: Supplementary file 1 — Supplementary Information. [file 41598_2024_63820_MOESM1_ESM.zip › Data/Tilandsias/Til2/ProfileCrop.tif]

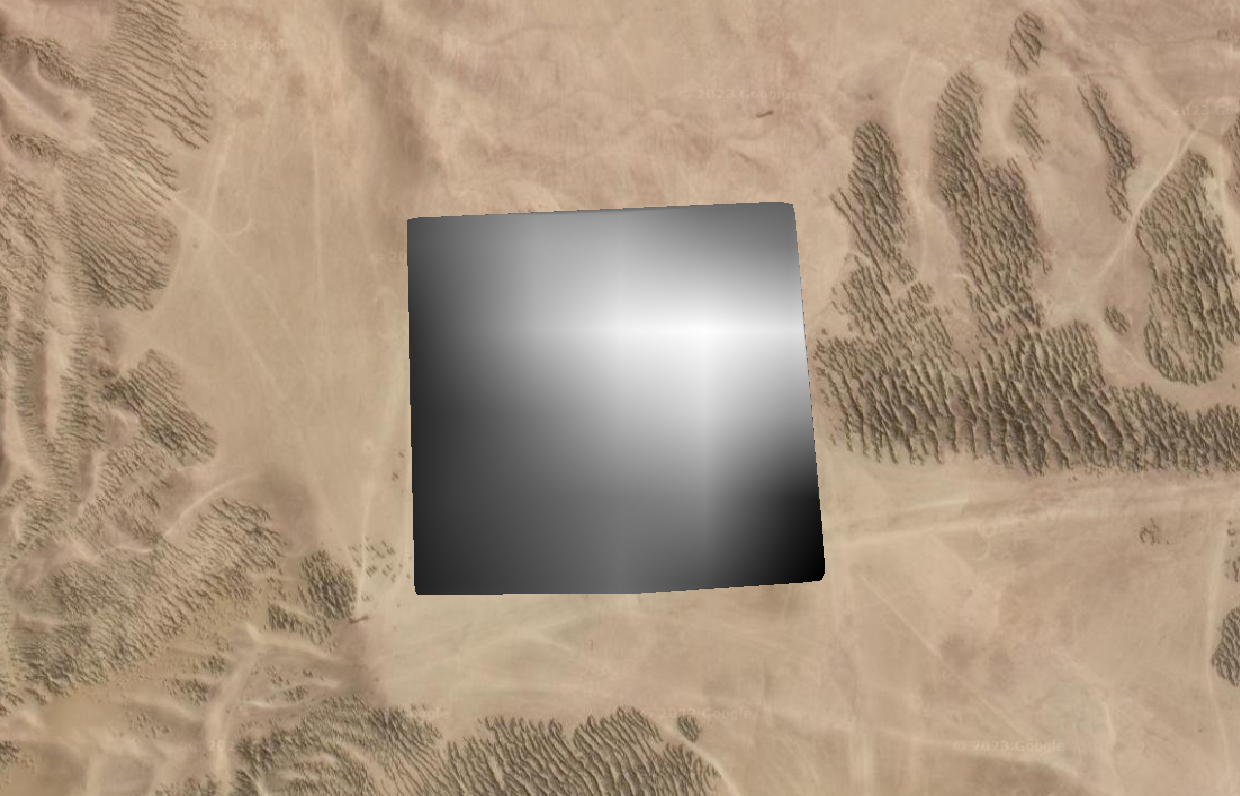

Supplement: Supplementary file 1 — Supplementary Information. [file 41598_2024_63820_MOESM1_ESM.zip › Data/Tilandsias/Til2/Profile.tif]

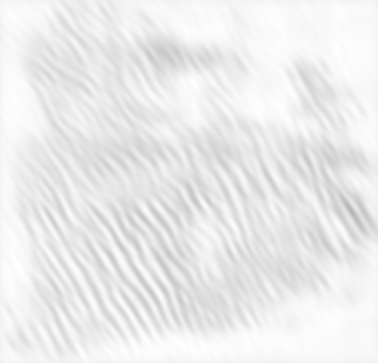

Supplement: Supplementary file 1 — Supplementary Information. [file 41598_2024_63820_MOESM1_ESM.zip › Data/Tilandsias/Til2/InvFFT.tif]

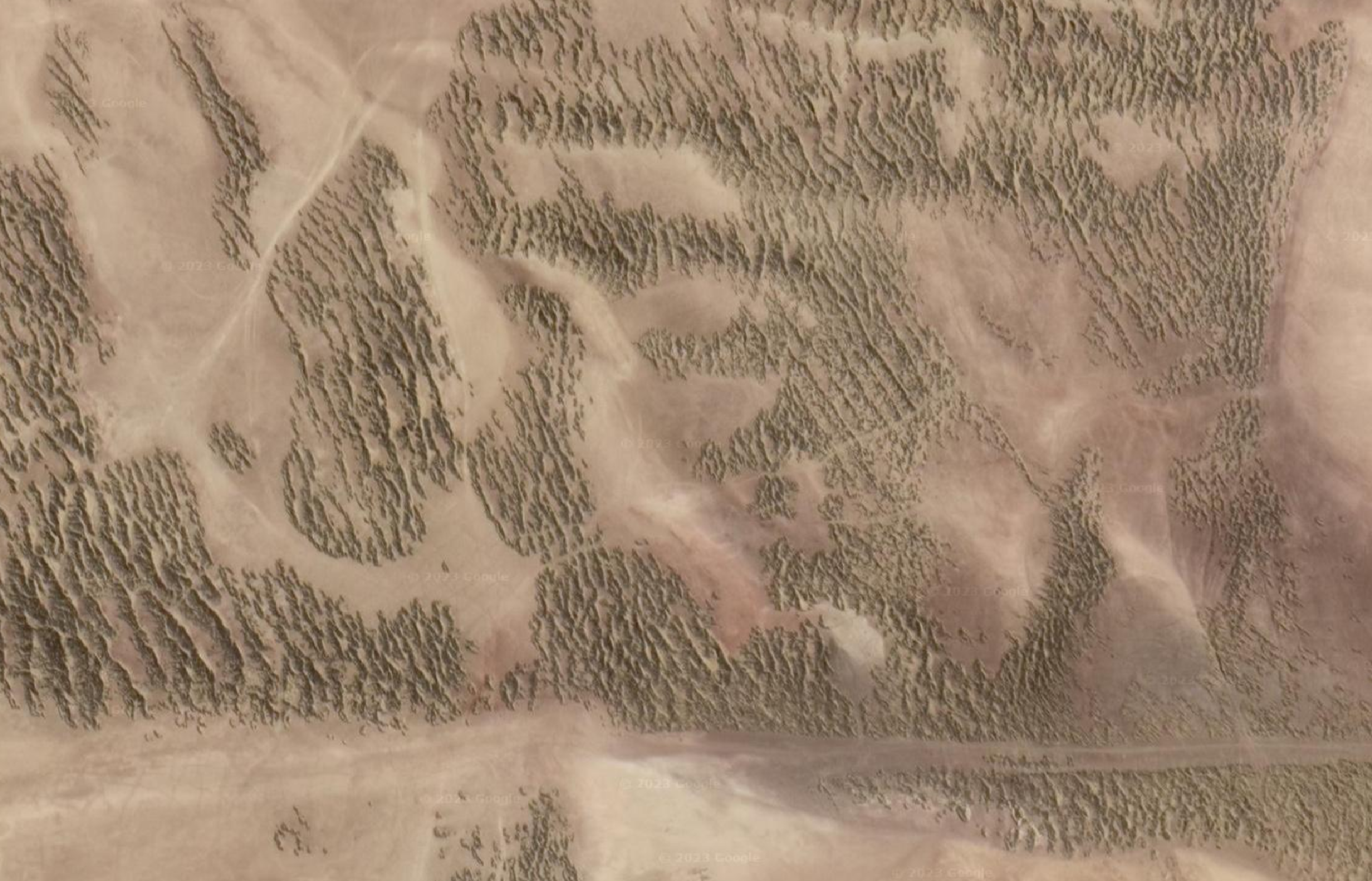

Supplement: Supplementary file 1 — Supplementary Information. [file 41598_2024_63820_MOESM1_ESM.zip › Data/Tilandsias/Til5/Patterm.tif]

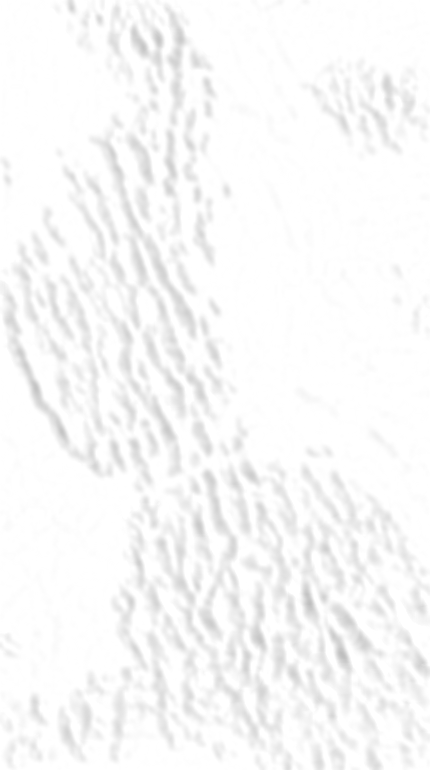

Supplement: Supplementary file 1 — Supplementary Information. [file 41598_2024_63820_MOESM1_ESM.zip › Data/Tilandsias/Til5/Filtered.tif]

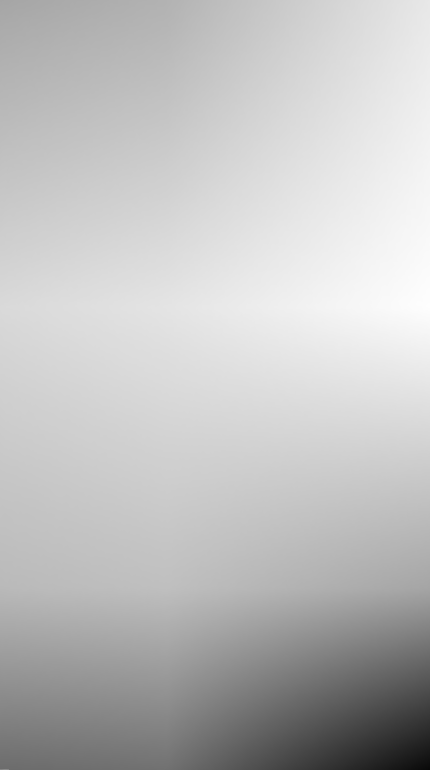

Supplement: Supplementary file 1 — Supplementary Information. [file 41598_2024_63820_MOESM1_ESM.zip › Data/Tilandsias/Til5/ProfileCrop.tif]

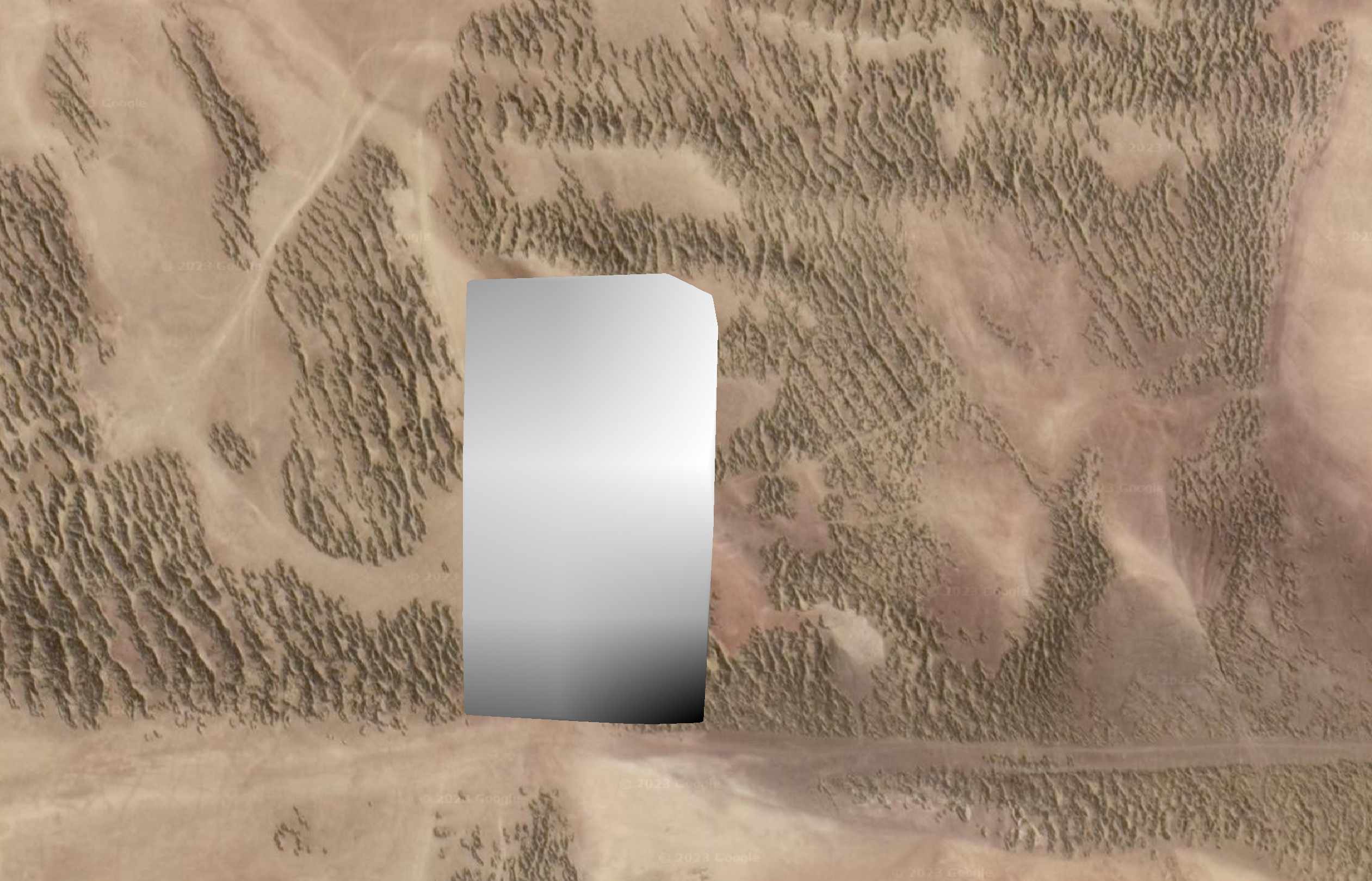

Supplement: Supplementary file 1 — Supplementary Information. [file 41598_2024_63820_MOESM1_ESM.zip › Data/Tilandsias/Til5/Profile.tif]

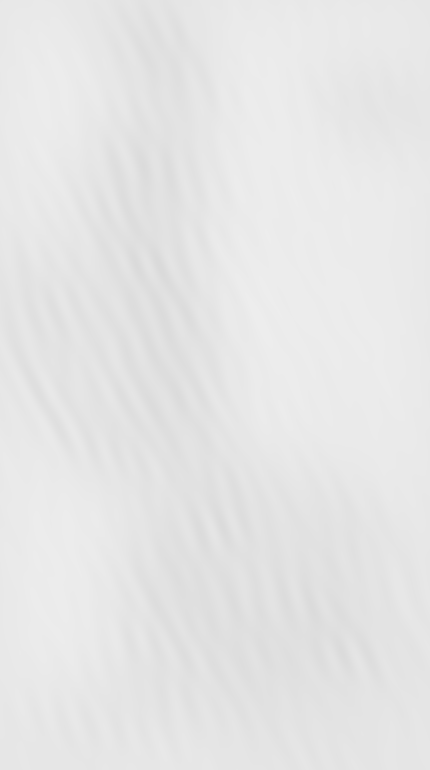

Supplement: Supplementary file 1 — Supplementary Information. [file 41598_2024_63820_MOESM1_ESM.zip › Data/Tilandsias/Til5/InvFFT.tif]

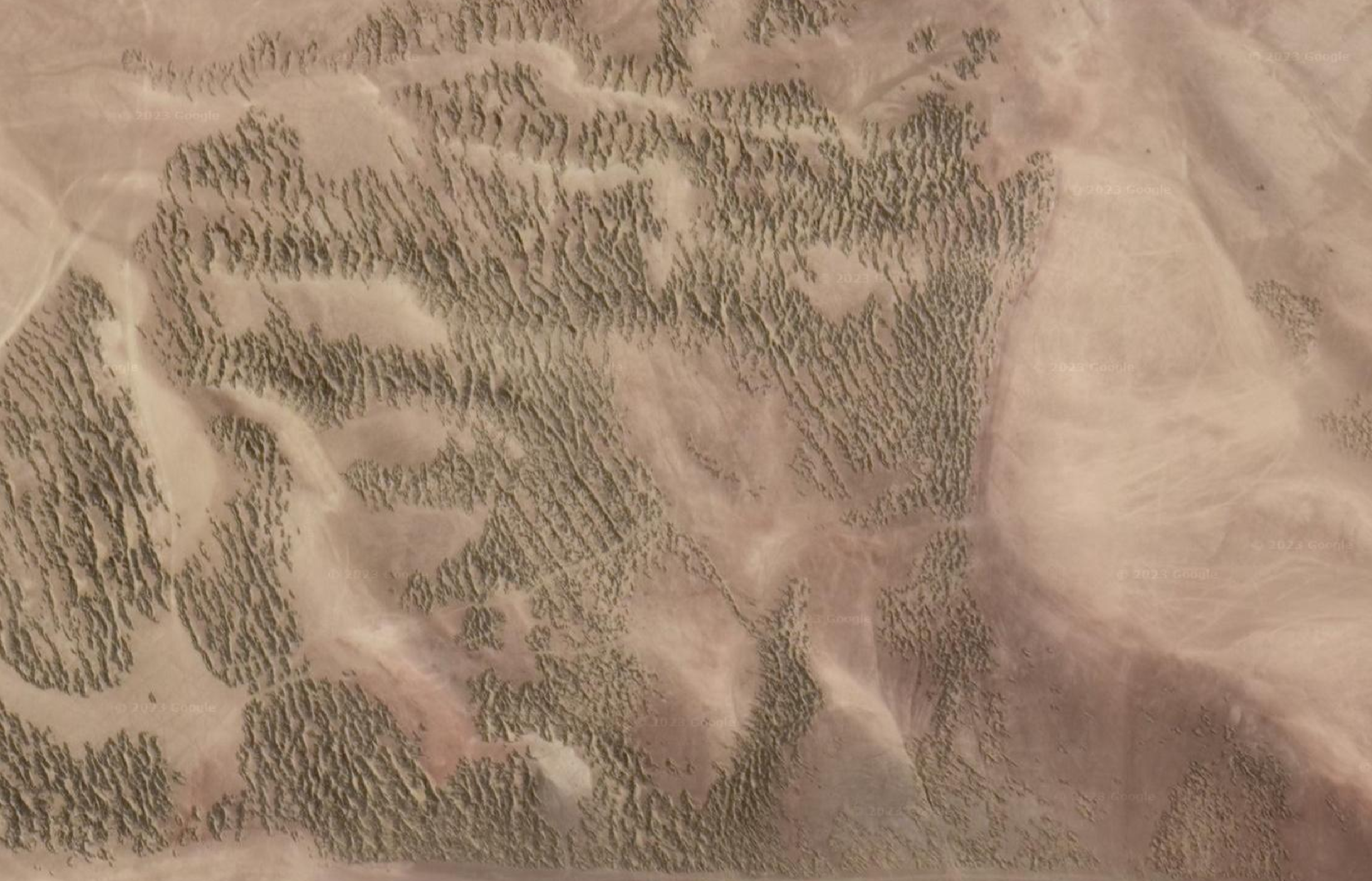

Supplement: Supplementary file 1 — Supplementary Information. [file 41598_2024_63820_MOESM1_ESM.zip › Data/Tilandsias/Til9/Pattern.tif]

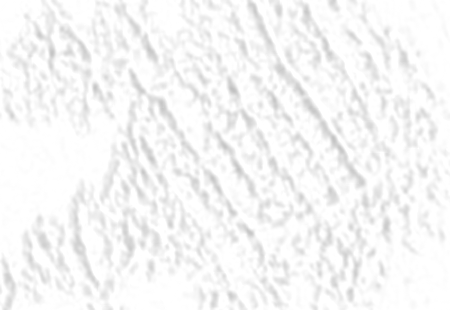

Supplement: Supplementary file 1 — Supplementary Information. [file 41598_2024_63820_MOESM1_ESM.zip › Data/Tilandsias/Til9/Filtered.tif]

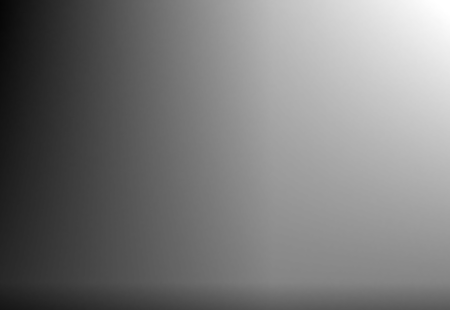

Supplement: Supplementary file 1 — Supplementary Information. [file 41598_2024_63820_MOESM1_ESM.zip › Data/Tilandsias/Til9/ProfileCrop.tif]

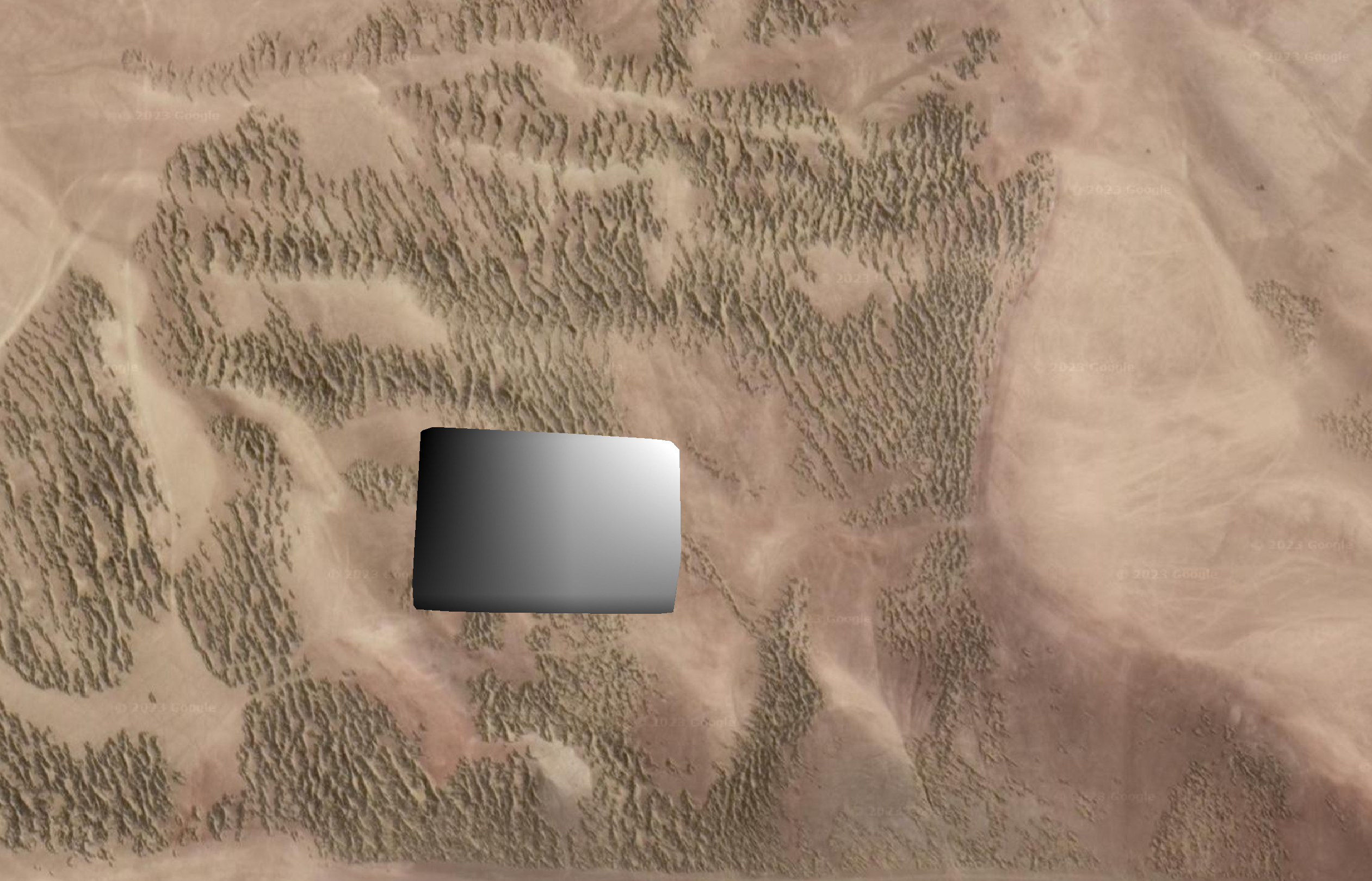

Supplement: Supplementary file 1 — Supplementary Information. [file 41598_2024_63820_MOESM1_ESM.zip › Data/Tilandsias/Til9/Profile.tif]

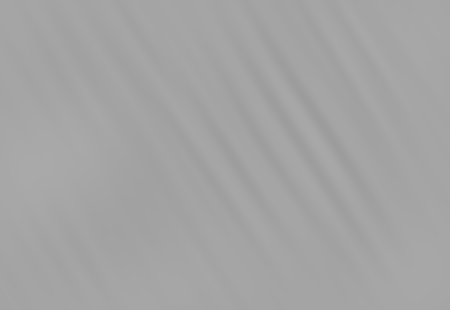

Supplement: Supplementary file 1 — Supplementary Information. [file 41598_2024_63820_MOESM1_ESM.zip › Data/Tilandsias/Til9/InvFFT.tif]

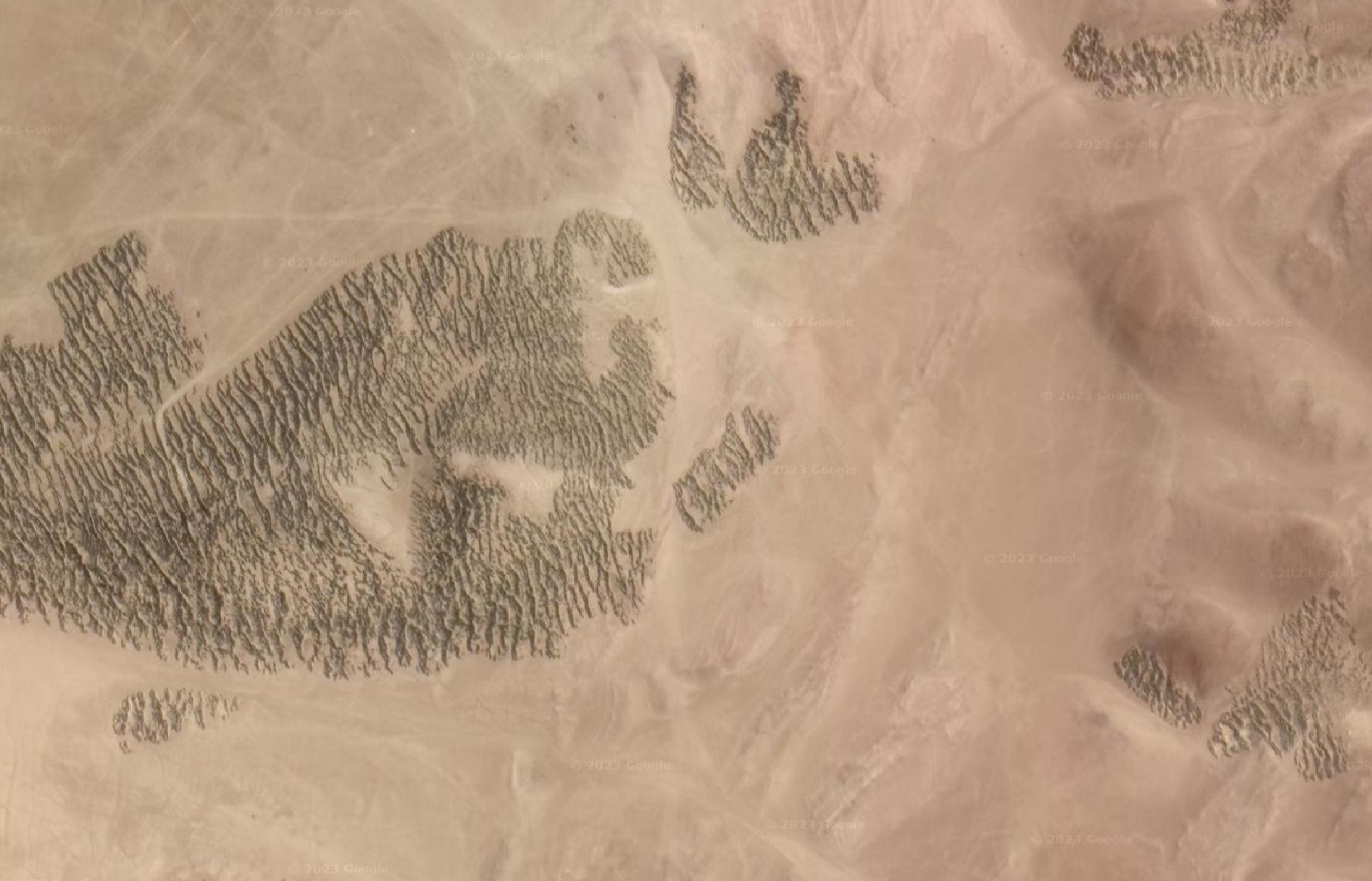

Supplement: Supplementary file 1 — Supplementary Information. [file 41598_2024_63820_MOESM1_ESM.zip › Data/Tilandsias/Til7/Pattern.tif]

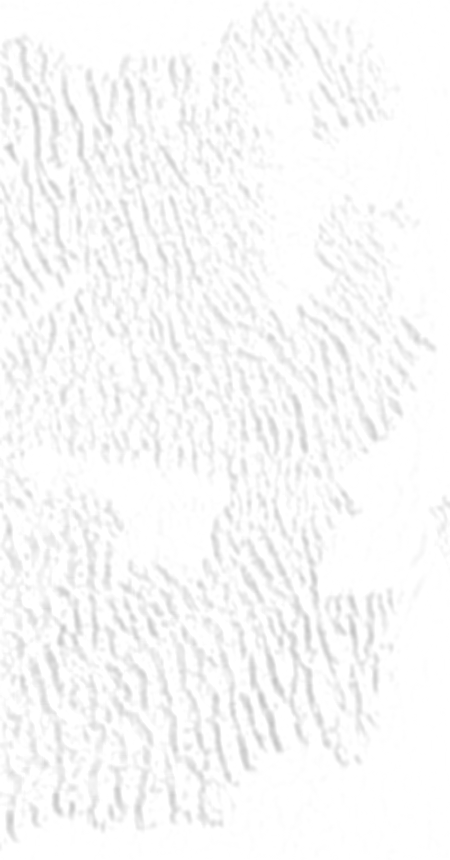

Supplement: Supplementary file 1 — Supplementary Information. [file 41598_2024_63820_MOESM1_ESM.zip › Data/Tilandsias/Til7/Filtered.tif]

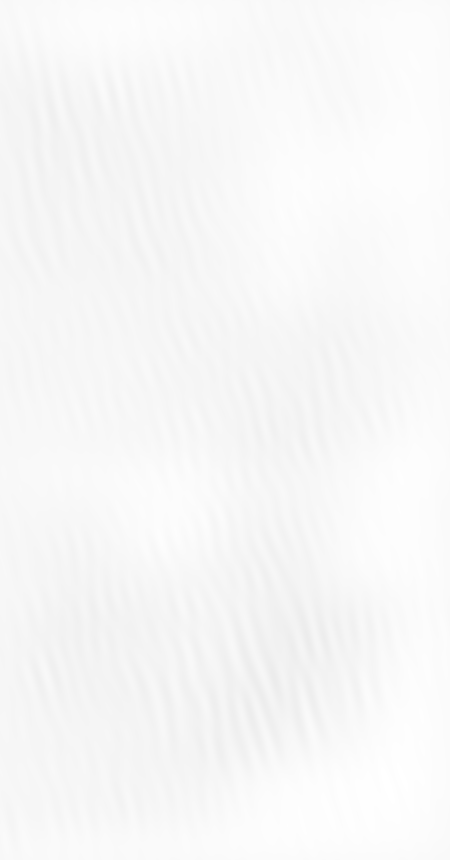

Supplement: Supplementary file 1 — Supplementary Information. [file 41598_2024_63820_MOESM1_ESM.zip › Data/Tilandsias/Til7/InveFFT.tif]

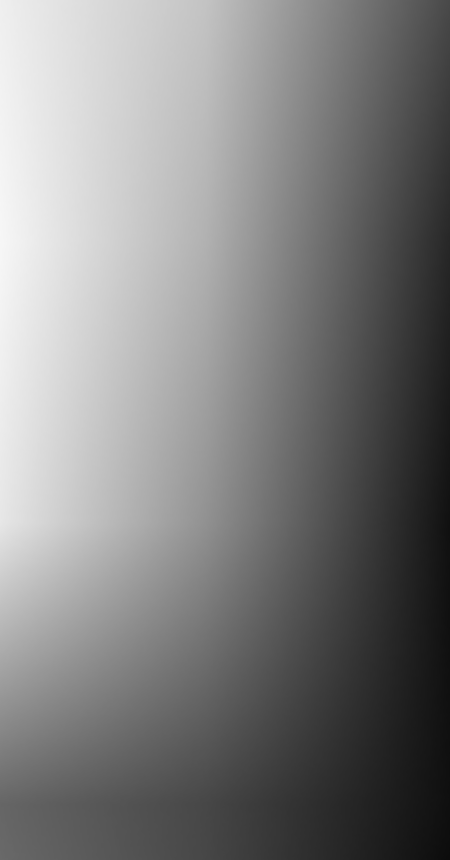

Supplement: Supplementary file 1 — Supplementary Information. [file 41598_2024_63820_MOESM1_ESM.zip › Data/Tilandsias/Til7/ProfileCrop.tif]

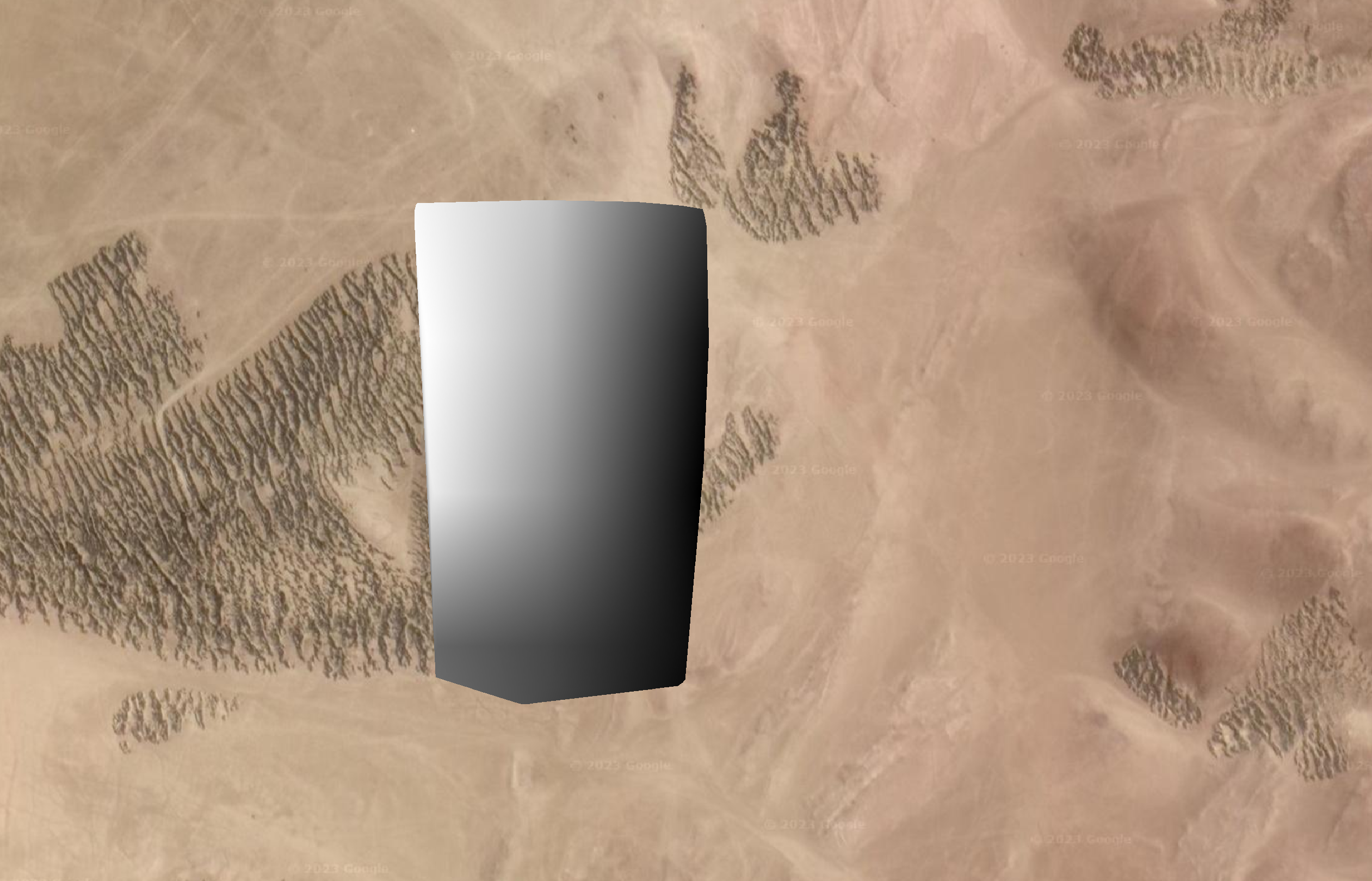

Supplement: Supplementary file 1 — Supplementary Information. [file 41598_2024_63820_MOESM1_ESM.zip › Data/Tilandsias/Til7/Profile.tif]

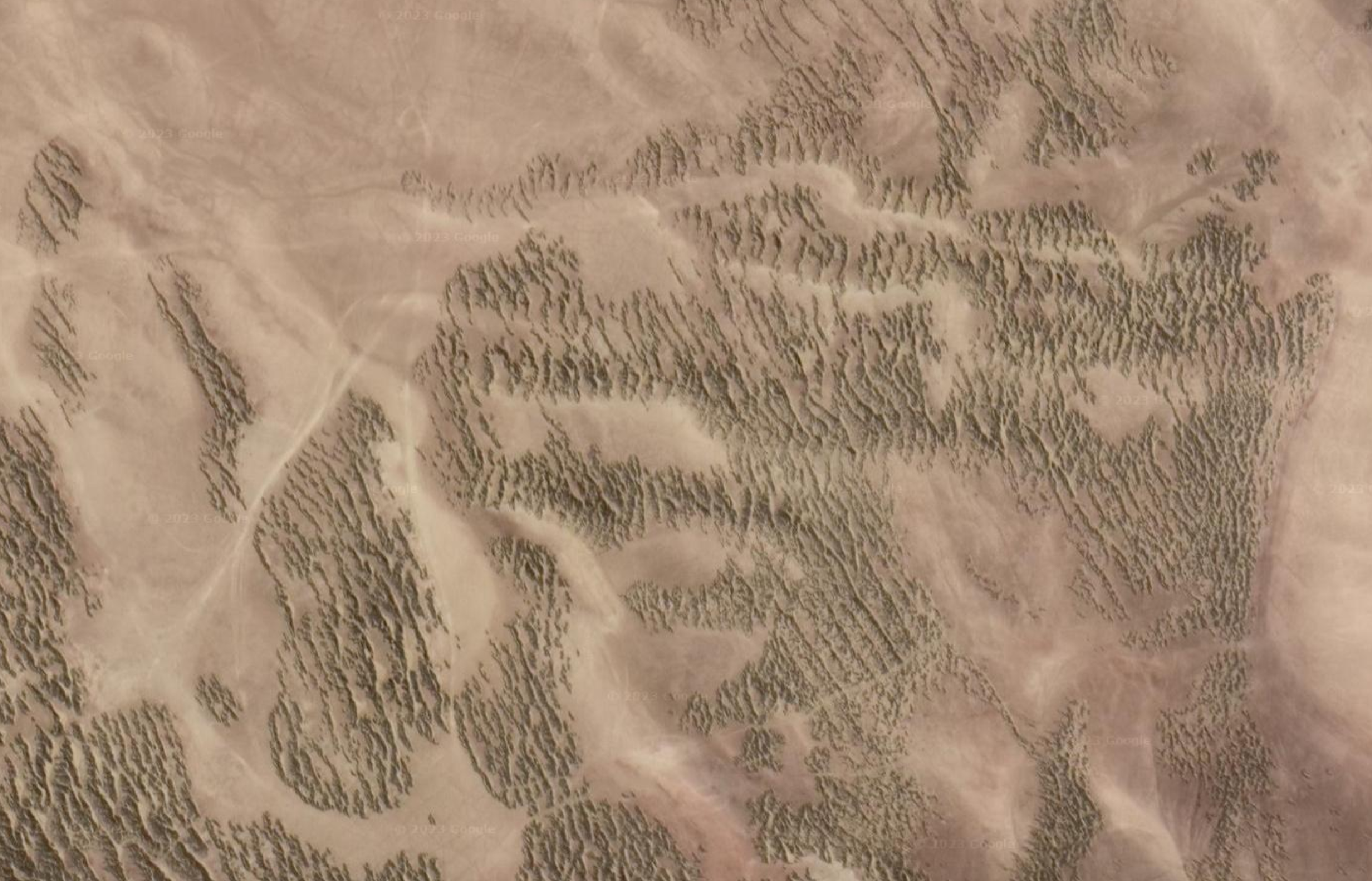

Supplement: Supplementary file 1 — Supplementary Information. [file 41598_2024_63820_MOESM1_ESM.zip › Data/Tilandsias/Til6/Pattern.tif]

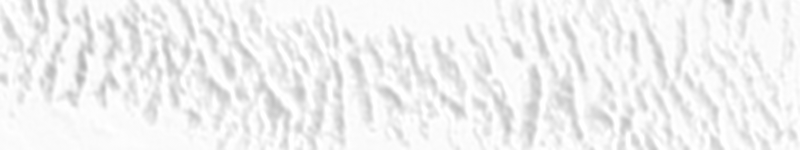

Supplement: Supplementary file 1 — Supplementary Information. [file 41598_2024_63820_MOESM1_ESM.zip › Data/Tilandsias/Til6/Filtered.tif]

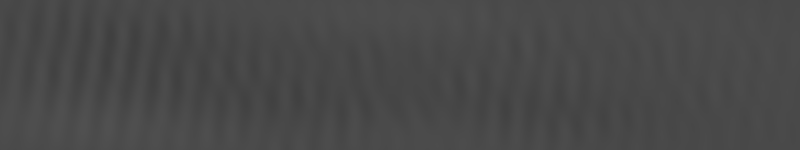

Supplement: Supplementary file 1 — Supplementary Information. [file 41598_2024_63820_MOESM1_ESM.zip › Data/Tilandsias/Til6/InveFFT.tif]

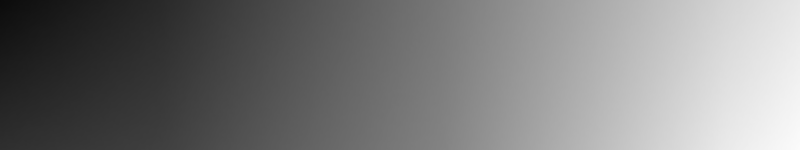

Supplement: Supplementary file 1 — Supplementary Information. [file 41598_2024_63820_MOESM1_ESM.zip › Data/Tilandsias/Til6/ProfileCrop.tif]

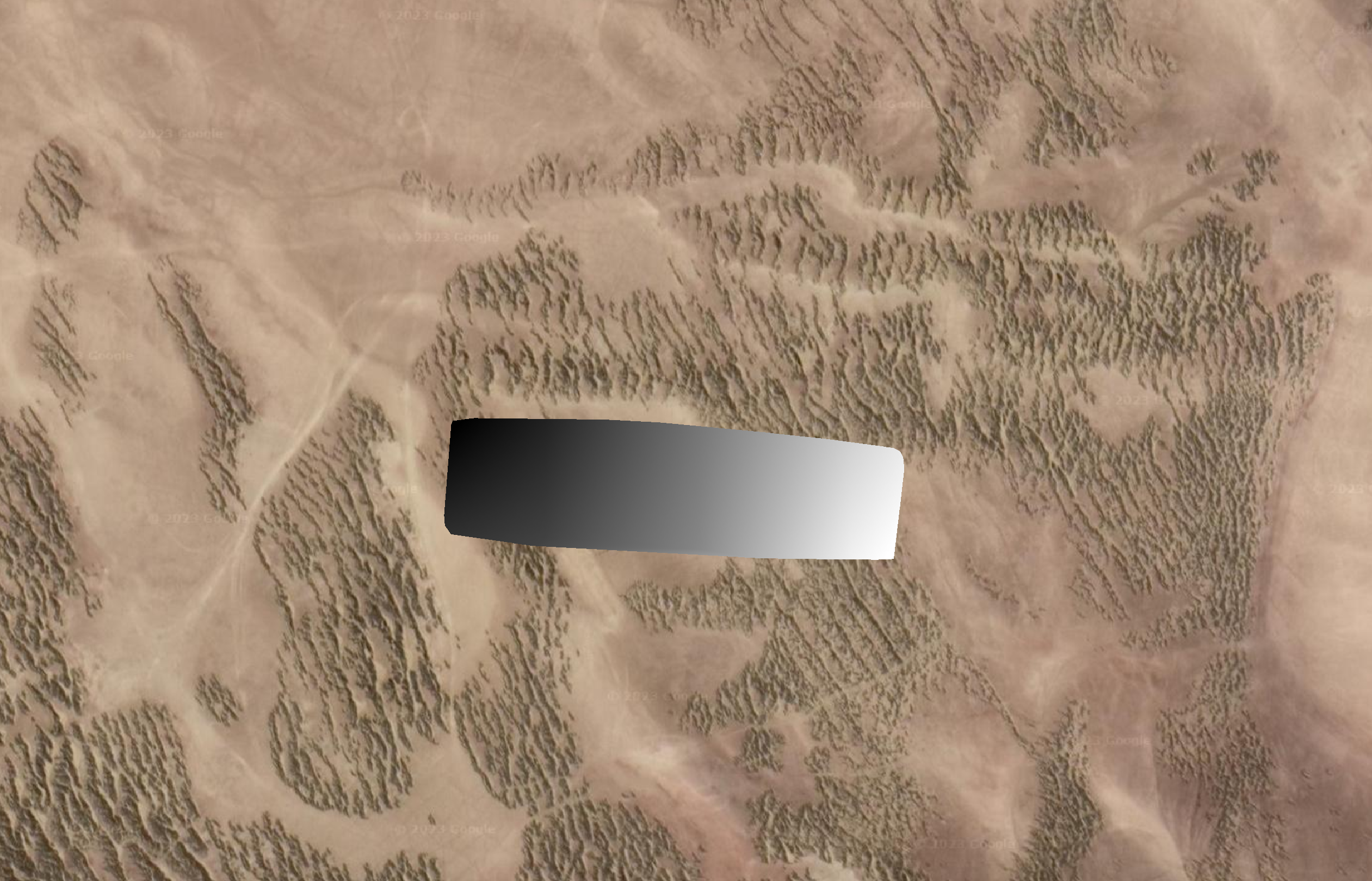

Supplement: Supplementary file 1 — Supplementary Information. [file 41598_2024_63820_MOESM1_ESM.zip › Data/Tilandsias/Til6/Profile.tif]

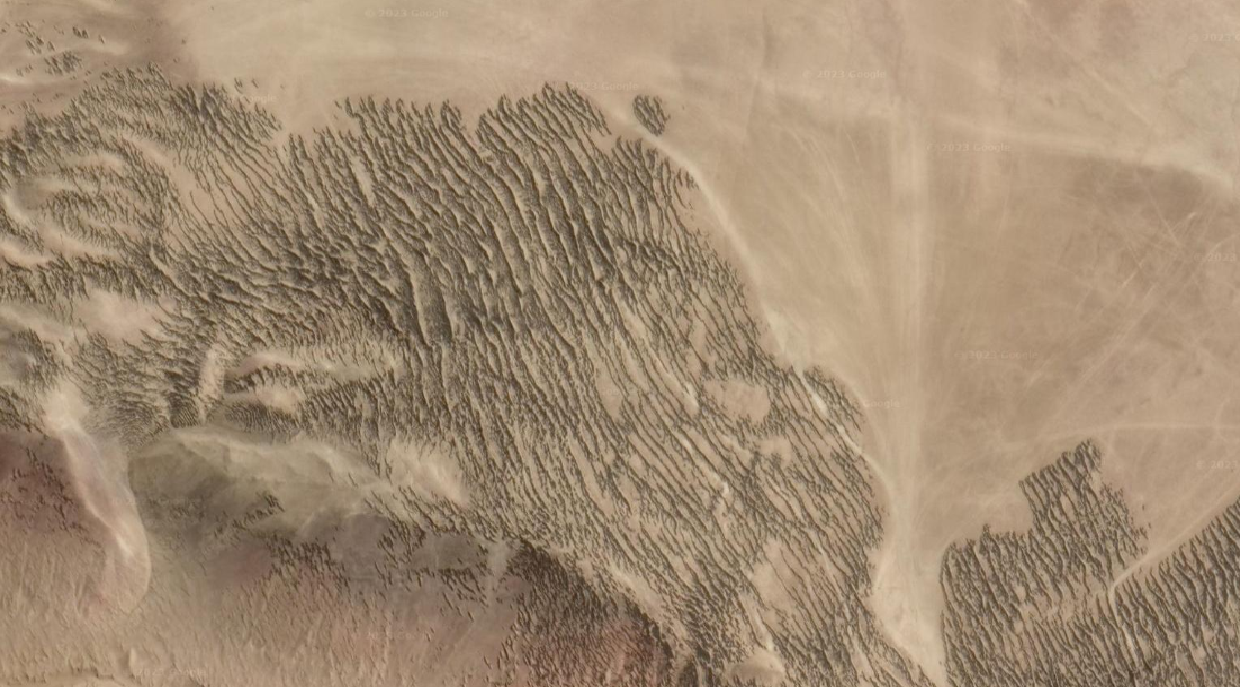

Supplement: Supplementary file 1 — Supplementary Information. [file 41598_2024_63820_MOESM1_ESM.zip › Data/Tilandsias/Til1/Pattern.tif]

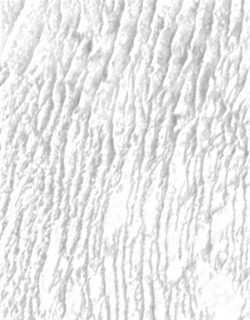

Supplement: Supplementary file 1 — Supplementary Information. [file 41598_2024_63820_MOESM1_ESM.zip › Data/Tilandsias/Til1/Filtered.tif]

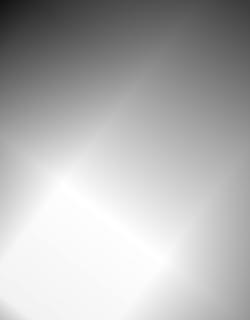

Supplement: Supplementary file 1 — Supplementary Information. [file 41598_2024_63820_MOESM1_ESM.zip › Data/Tilandsias/Til1/ProfileCrop.tif]

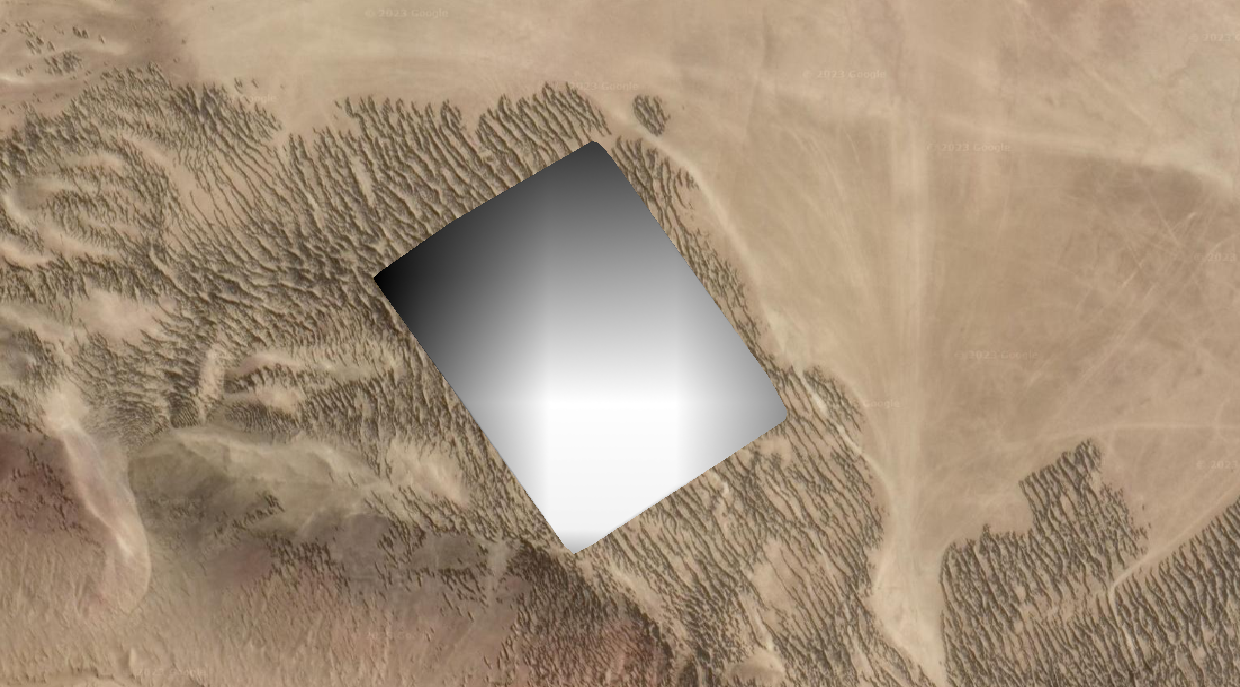

Supplement: Supplementary file 1 — Supplementary Information. [file 41598_2024_63820_MOESM1_ESM.zip › Data/Tilandsias/Til1/Profile.tif]

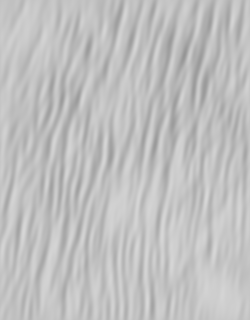

Supplement: Supplementary file 1 — Supplementary Information. [file 41598_2024_63820_MOESM1_ESM.zip › Data/Tilandsias/Til1/InvFFT.tif]

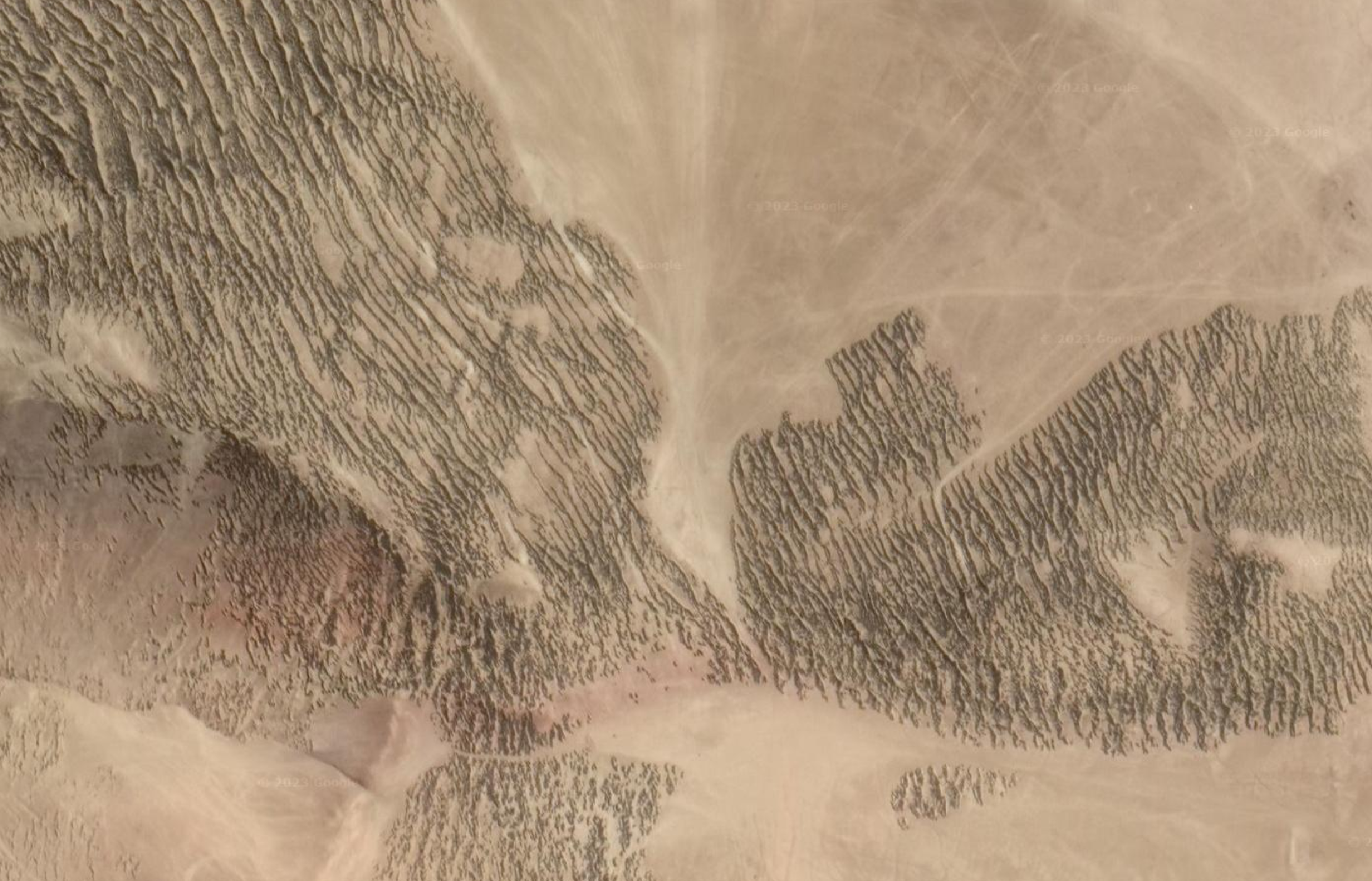

Supplement: Supplementary file 1 — Supplementary Information. [file 41598_2024_63820_MOESM1_ESM.zip › Data/Tilandsias/Til8/Pattern.tif]

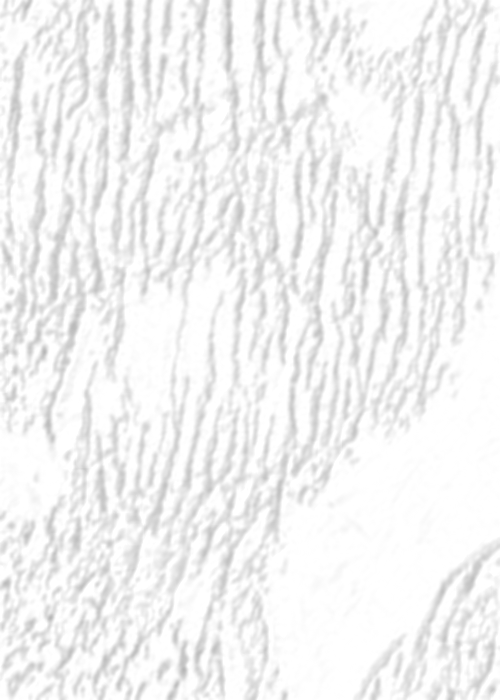

Supplement: Supplementary file 1 — Supplementary Information. [file 41598_2024_63820_MOESM1_ESM.zip › Data/Tilandsias/Til8/Filtered.tif]

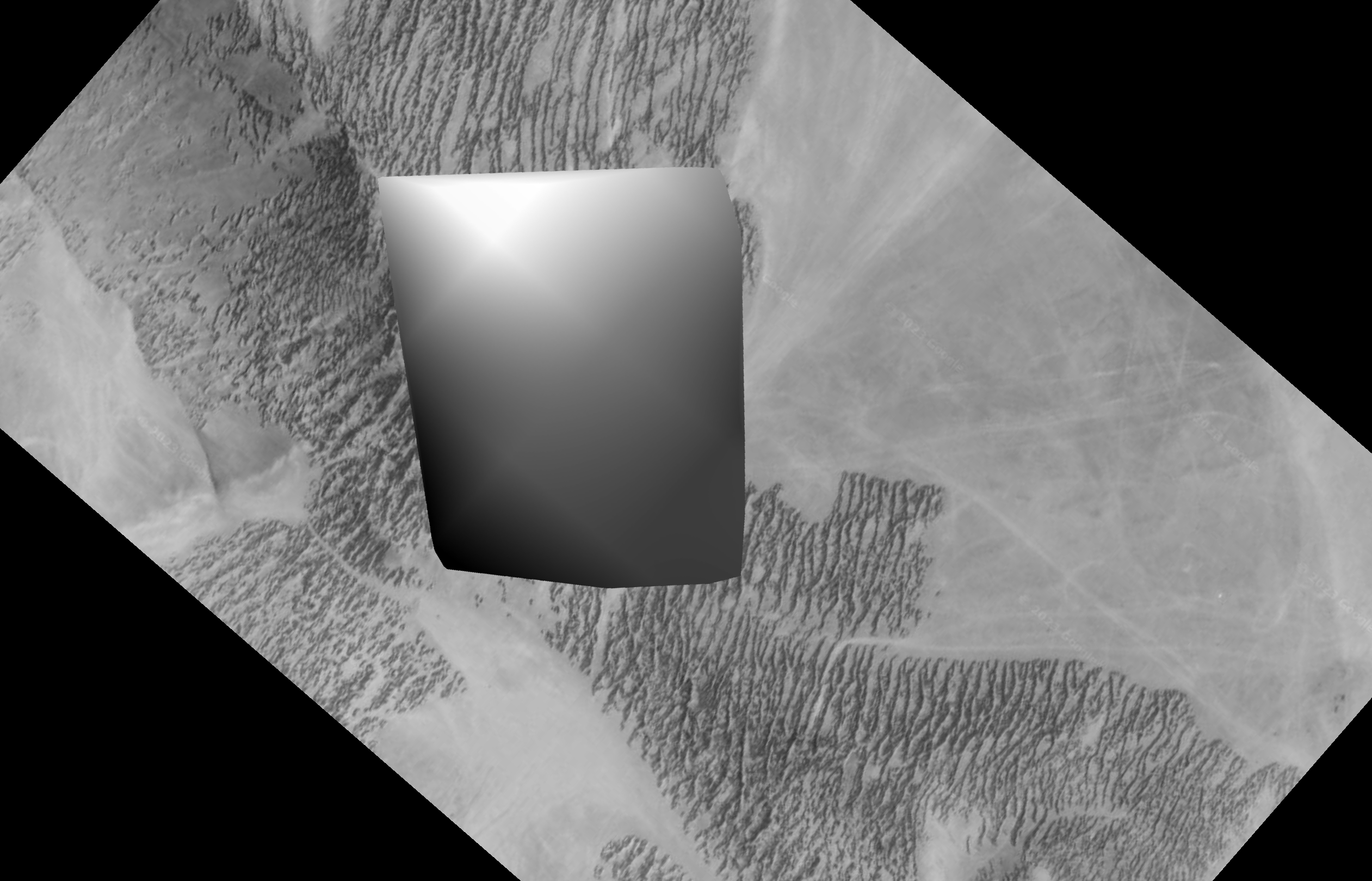

Supplement: Supplementary file 1 — Supplementary Information. [file 41598_2024_63820_MOESM1_ESM.zip › Data/Tilandsias/Til8/RotProfile.tif]

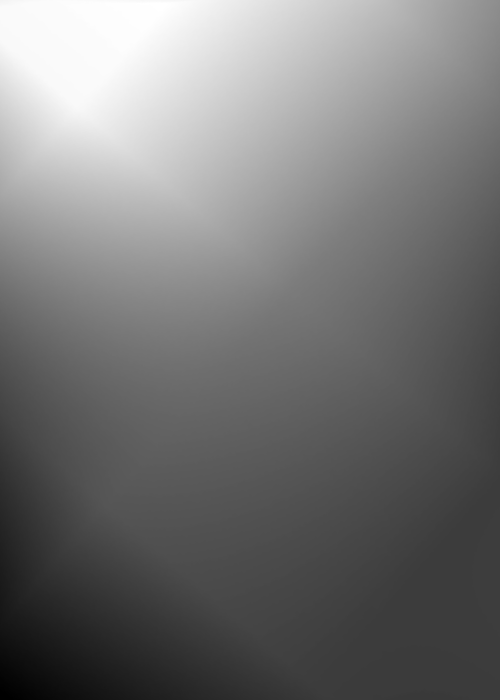

Supplement: Supplementary file 1 — Supplementary Information. [file 41598_2024_63820_MOESM1_ESM.zip › Data/Tilandsias/Til8/ProfileCrop.tif]

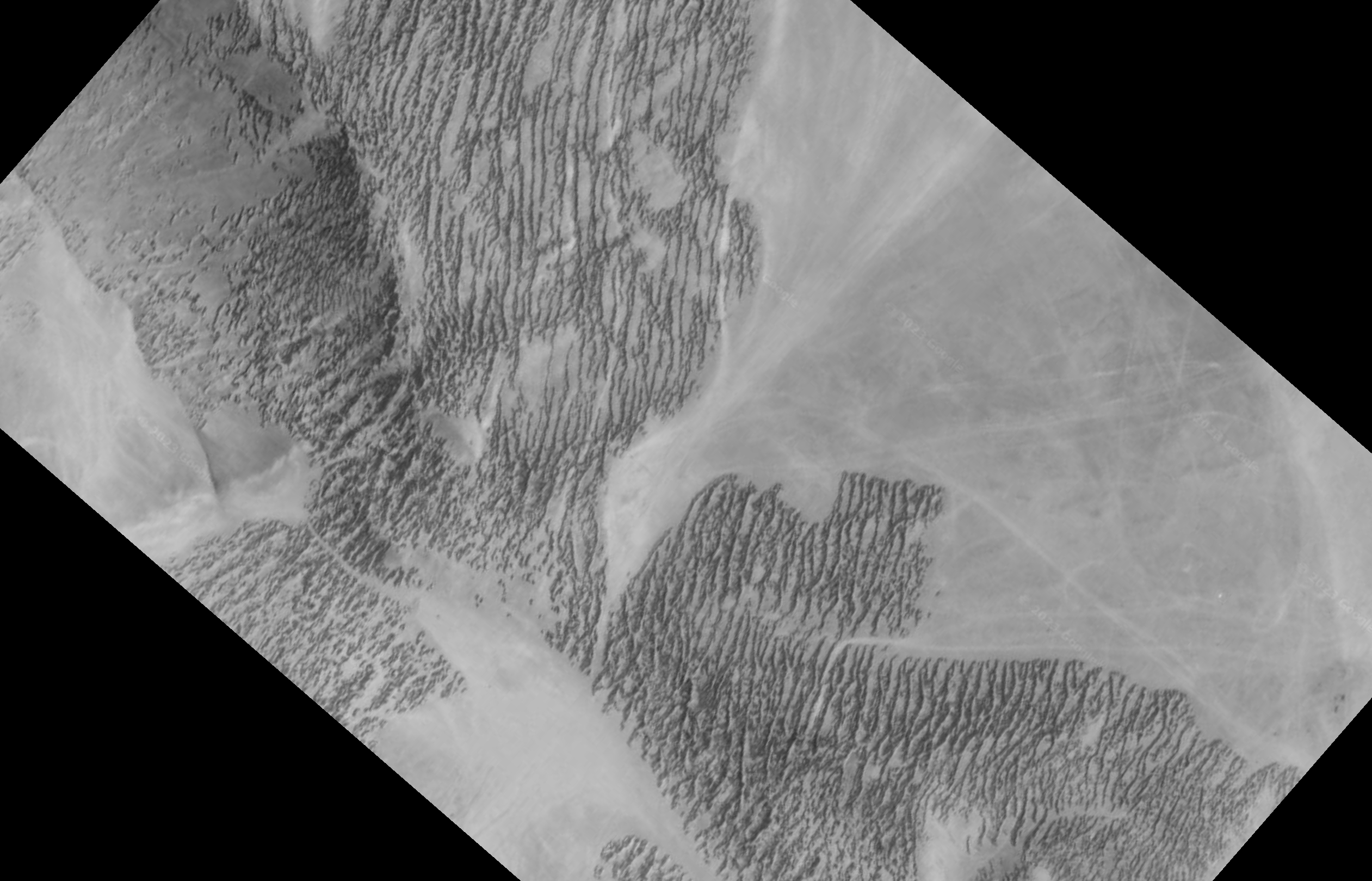

Supplement: Supplementary file 1 — Supplementary Information. [file 41598_2024_63820_MOESM1_ESM.zip › Data/Tilandsias/Til8/RotPatt.tif]

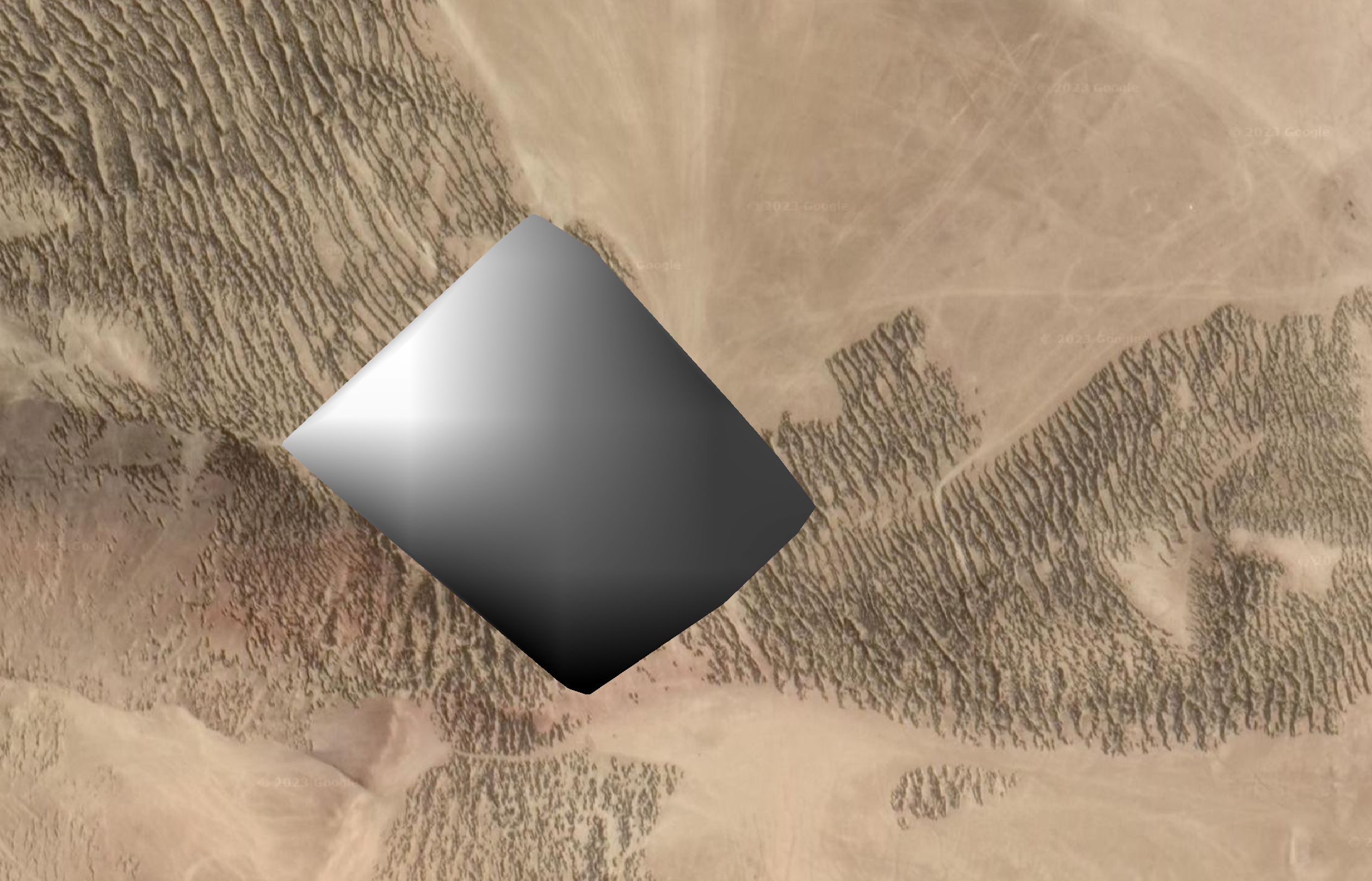

Supplement: Supplementary file 1 — Supplementary Information. [file 41598_2024_63820_MOESM1_ESM.zip › Data/Tilandsias/Til8/Profile.tif]

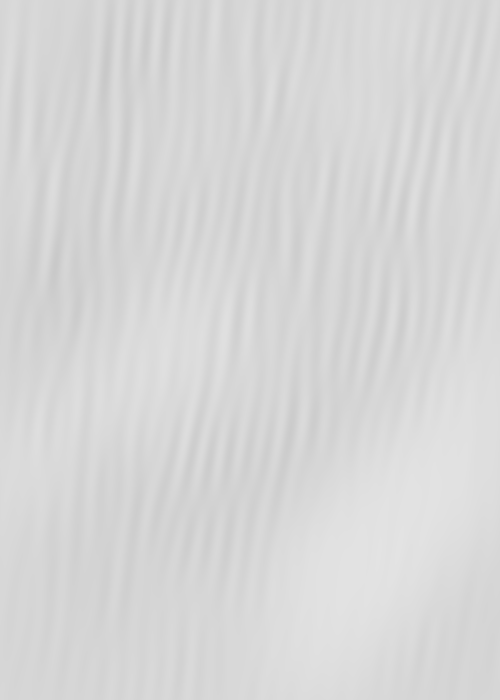

Supplement: Supplementary file 1 — Supplementary Information. [file 41598_2024_63820_MOESM1_ESM.zip › Data/Tilandsias/Til8/InvFFT.tif]
